# Impact of Pomegranate on Probiotic Growth, Viability, Transcriptome and Metabolism

**DOI:** 10.3390/microorganisms11020404

**Published:** 2023-02-05

**Authors:** Sarah O’Flaherty, Natalia Cobian, Rodolphe Barrangou

**Affiliations:** Department of Food, Bioprocessing & Nutrition Sciences, North Carolina State University, Raleigh, NC 27695, USA

**Keywords:** probiotic, prebiotic, pomegranate, lactobacilli, transcriptomic

## Abstract

Despite rising interest in understanding intestinal bacterial survival in situ, relatively little attention has been devoted to deciphering the interaction between bacteria and functional food ingredients. Here, we examined the interplay between diverse beneficial *Lactobacillaceae* species and a pomegranate (POM) extract and determined the impact of this functional ingredient on bacterial growth, cell survival, transcription and target metabolite genesis. Three commercially available probiotic strains (*Lactobacillus acidophilus* NCFM, *Lacticaseibacillus rhamnosus* GG and *Lactiplantibacillus plantarum* Lp-115) were used in growth assays and flow cytometry analysis, indicating differential responses to the presence of POM extract across the three strains. The inclusion of POM extract in the growth medium had the greatest impact on *L. acidophilus* cell counts. LIVE/DEAD staining determined significantly fewer dead cells when *L. acidophilus* was grown with POM extract compared to the control with no POM (1.23% versus 7.23%). Whole-transcriptome analysis following exposure to POM extract showed markedly different global transcriptome responses, with 15.88% of the *L. acidophilus* transcriptome, 19.32% of the *L. rhamnosus* transcriptome and only 2.37% of the *L. plantarum* transcriptome differentially expressed. We also noted strain-dependent metabolite concentrations in the medium with POM extract compared to the control medium for punicalagin, ellagic acid and gallic acid. Overall, the results show that POM extract triggers species-specific responses by probiotic strains and substantiates the rising interest in using POM as a prebiotic compound.

## 1. Introduction

Many foods encompass active ingredients, and functional foods have been known for centuries to exert health benefits, although the molecular basis of their modes of action remains undeciphered [1]. Functional foods can provide health benefits beyond their nutritional value and are an ever-increasing research area, as the industry was reported to be worth USD 173.26 billion in 2019 and is estimated to grow 7.5% annually, increasing to USD 309 billion by 2027 [2]. In addition, the growth of the functional food industry is fueled by more awareness of the food we consume and reported health benefits that can be achieved through natural means. Indeed, there has been an explosion of studies of the microbiome in the gastrointestinal tract (GIT) and increased examination of the importance of this microbial community on both health and disease [3,4,5]. Therefore, understanding the interaction and relationship between functional foods and beneficial bacteria is imperative to decipher their function in the GIT [6]. In fact, the National Institute of Health launched a strategic plan in 2021 entitled “Mapping a pathway to research on whole person health”, which includes multisystem approaches to studying human health, including a nutritional approach encompassing probiotics, prebiotics, phytochemicals, dietary plants, and food and microbiome metabolites, amongst others [7].

Pomegranate (POM), a fruit consumed for centuries [8], is considered a functional food given its wide range of documented beneficial effects, including antibacterial and anticarcinogenic, as well as skin protection from UV photodamage [9,10,11,12]. In addition to its nutritional attributes, pomegranate is a rich source of plant metabolites, including anthocyanins and polyphenols such as ellagic acid and punicalagins, which are further metabolized by the microbiota in the GIT into bioactive metabolites [12]. Studies have shown that many of the health benefits of POM are due to ellagitannins, particularly punicalagins that are hydrolyzed into ellagic acid [11]. This hydrolysis initially occurs in the small intestine, where ellagic acid is absorbed, and the bacteria in the large intestine metabolize any remaining ellagitannins and ellagic acid into urolithins. In recent studies, punicalagin, the most abundant polyphenol in POM, has demonstrated antiviral [13,14], antibacterial [15] and anticancer properties and has been revealed to play a potential role in preventing cardiovascular disease [11]. The activity of urolithins, which are GIT metabolites, includes anticancer and anti-inflammatory properties [16,17]. The antibacterial effects of POM have been shown against various pathogenic bacteria, while intriguingly enhancing the cell numbers of some beneficial bacteria, including probiotics [18,19,20,21]. Probiotics are defined as “live microorganisms that, when administered in adequate amounts, confer a health benefit on the host” [22]. Probiotic bacteria have shown numerous and wide-ranging beneficial effects on the host, particularly in the GIT, including the exclusion of pathogens, immunomodulation, nutrient turnover and the modulation of bile acids [23]. For consideration as a probiotic, strains should meet certain criteria and are often members of genera associated with the human gut microbiome, such as *Bifidobacterium* and Lactobacillaceae [9]. Previous studies have shown a prebiotic effect of POM extract and POM juice on lactobacilli in vitro, with an increase in cell counts and the utilization of ellagic acid [20]. In addition, the consumption of POM extract by healthy volunteers over a 4-week period led to changes in the gut microbiome and levels of fecal urolithin A [24]. In the urolithin A-producer group, there was in increase in the detection of *Lactobacillus* after 4 weeks compared to the baseline [24]. 

Considering the potential health benefits of POM extract, we studied the growth of *Lactobacillus* species in the presence of POM extract and used RNA sequencing to determine the whole-transcriptome response of three established commercial probiotic strains. We also performed targeted metabolite analysis to further consider the prebiotic potential of POM extract on commercial probiotic strains.

## 2. Materials and Methods

### 2.1. Preparation of Pomegranate Extract and Growth Media

In this study, the starting material was pomegranate polyphenol extract dietary supplement (POMx) powder from POM Wonderful, Inc., Los Angeles, CA. Details of the preparation of the pomegranate polyphenol extract dietary supplement (POMx) are described by Herber et al., 2007 [25]. Briefly, after pressing to obtain juice, the aqueous portion is extracted from the fruit residue and seeds are removed, and then a powder is produced by solid-phase extraction of the liquid concentrate [25]. This group also reported the polyphenol content of POMx to be 61% gallic acid equivalents, of which 77% are oligomers of gallic acid, ellagic acid and glucose, 19% are ellagitannins (punicalagins and punicalins) and 4% are free ellagic acid [25]. In addition, they reported that POMx contains 3.3% moisture, 2.2% ash, 2.9% sugars, 1.9% organic acids and 0.7% nitrogen. The preparation of the pomegranate (POM) extract that was used in these studies from POMx was followed as described previously [26,27]. The POM extract was dissolved in ultrapure water (Life Technologies, Carlsbad, CA, USA) at a concentration of 7 mg/mL and vortexed for 10 min. The solution was then centrifuged at 1717× *g* for 10 min and filter-sterilized through a 0.45µm filter. Aliquots of the stock solution were stored at −20 °C. A semi-defined medium (SDM; composition details can be found in Supplementary Materials) was used as the base medium [28], with the addition of glucose (1% (for transfers) or 0.5% (for experiments)). In this study, “transfers” refers to introducing the strains to the growth medium prior to experiments. The stock POM extract was diluted in SDM to 400 µg/mL for transfers and 400 µg/mL (P400) or 800 µg/mL (P800) for growth assays. All subsequent experiments (Figure S1) were performed with a concentration of P400, given that the initial results of growth assays indicated that the higher concentration resulted in potential strain inhibition. 

### 2.2. Growth Assays

*L. acidophilus* NCFM (Lac), *L. rhamnosus* GG (ATCC 53103, Lrh) and *L. plantarum* Lp-115 (Lpl) were grown from −80 °C glycerol stocks and transferred (1% inoculum) in SDM medium (1% glucose, with or without P400) for two overnight transfers at 37 °C prior to growth assays (1% inoculum) in test tubes with growth medium without POM (control) or with P400 or P800 to determine the OD and CFU/mL over a 24 h time period. OD 600 mm values were determined in cuvettes using a spectrometer (Genesys 20, ThermoSpectronic) at T0, T4, T6, T8, T12 and T24 h. The CFU/mL values were determined at T0, T8 and T24 h by performing serial dilutions and plating on MRS agar plates. 

### 2.3. Flow Cytometry Analysis

Flow cytometry was performed as described previously [29]. *L. acidophilus* NCFM, *L. plantarum* Lp-115 and *L. rhamnosus* GG were transferred twice in SDM medium and grown for 24 h in SDM medium (control) or transferred twice in SDM medium with P400 and grown for 24 h in SDM medium with P400 (test) at 37 °C prior to flow cytometry analysis (Figure S1). Bacterial cultures were then centrifuged at 1717× *g* for 10 min, washed and resuspended in phosphate-buffered saline (Life Technologies, Carlsbad, CA, USA). A CytoFLEX Flow Cytometer instrument (Beckman Coulter, Brea, CA, USA) located at the College of Veterinary Medicine Flow Cytometry and Cell Sorting core facility (North Carolina State University) was used to determine the scattering patterns. LIVE/DEAD cell viability staining was performed using propidium iodide as detailed by the manufacturer (Fisher Scientific, Waltham, MA, USA). Data analysis was performed with the CytExpert software (Beckman Coulter, Brea, CA, USA).

### 2.4. RNA Isolation and mRNA Sequencing and Data Analysis

*L. acidophilus* NCFM, *L. plantarum* Lp-115 and *L. rhamnosus* GG (two biological replicate cultures) were transferred twice in SDM (1% glucose no POM) or SDM (1% glucose and P400) and then grown to the mid-log phase (OD600 = 0.5–0.7) in SDM (0.5% glucose) broth or SDM (0.5% glucose and P400) at 37 °C under ambient atmospheric conditions. Cells were pelleted and flash-frozen, and pellets were stored at −80 °C. Methods for RNA isolation and RNA sequence analysis were as described previously [30]. Total RNA was extracted using the Zymo Direct-zol RNA MiniPrep kit (Zymo Research, Irvine, CA, USA). DNA was removed by incubating samples with Turbo DNase as described by the manufacturer (Ambion Inc., Austin, TX, USA), purified using the RNA clean and concentrator kit (Zymo Research) and checked for integrity by capillary electrophoresis on the Agilent Bioanalyzer (Agilent Technologies, Santa Clara, CA, USA).

Library preparation and sequencing were performed at The High-Throughput Sequencing and Genotyping Unit of the Roy J. Carver Biotechnology Center, University of Illinois at Urbana-Champaign. The Ribozero Bacteria kit (Illumina, San Diego, CA, USA) was used to remove rRNA, followed by library preparation with the TruSeq Stranded mRNAseq Sample Prep kit (Illumina). Libraries were then quantitated via qPCR and sequenced on one lane for 151 cycles from each end of the fragments on a NovaSeq 6000 using a NovaSeq SP reagent kit (Illumina); paired-end reads were 150 nts in length. Fastq files were generated and de-multiplexed with the bcl2fastq v2.20 Conversion Software (Illumina). Subsequent processes were performed with Geneious Prime [31]. For each strain, differential gene expression analysis was performed to compare gene expression between cells grown in the control (SDM) and test media (SDM with POM extract). Genes were considered significantly differentially expressed with a Log2 fold change of ±2 and a *p* value ≤ 0.05.

### 2.5. Preparation of Samples for Targeted Metabolite Detection

*L. acidophilus* NCFM, *L. plantarum* Lp-115 and *L. rhamnosus* GG were transferred twice in SDM (1% glucose), and cells were inoculated (1%) into SDM (0.5% glucose, control) or SDM (0.5% glucose with P400) and grown for 24 h at 37 °C. After 24 h, cultures were centrifuged at 2152× *g* for 3 min, and the cell-free supernatant was transferred to tubes for storage at −80 °C. Triplicate samples were prepared for each condition, including the control media at T0 and T24. Targeted detection of the metabolites punicalagin (Sigma, St Louis, MO, USA), ellagic acid (Sigma), gallic acid (Toronto Research Chemicals, North York, ON, CA), urolithin A (Sigma), urolithins C and D (Toronto Research Chemicals) was performed at the Molecular Education, Technology and Research Innovation Center (METRIC) core facility at NCSU. 

Cell-free supernatants were centrifuged (10,000× *g*, 5 min, 4 °C) and diluted (25 µL supernatant, 225 µL H_2_O) prior to analysis using an ultra-performance liquid chromatography–tandem mass spectrometer (UPLC-MS/MS). The analysis was performed using a Thermo Vanquish LC instrument (Thermo Fisher Scientific, San Jose, CA) coupled to a Thermo TSQ Altis triple quadrupole mass spectrometer (Thermo Fisher Scientific) with a heated electrospray ionization (HESI) source. Chromatographic separation was achieved on a Restek Raptor C18 column (2.1 *×* 100 mm, 1.8 mM) maintained at 30 °C. The following linear gradient of mobile phase A (H_2_O + 0.1% FA) and mobile phase B (MeCN + 0.1% FA) was used: 0–0.25 min (5%B, 0.2 mL/min), 0.25–6 min (5–30%B, 0.2 mL/min), 6–10 min (30–95%B, 0.2 mL/min), 10–12.5 min (95%B, 0.2 mL/min) and 12.51–15 min (5%B, 0.2 mL/min). Stock solutions (1 mg/mL) were prepared in DMSO or MeOH, and then individual stocks were combined and diluted with water to provide calibration curves in the linear range for each analyte (12.5–5000 ng/mL for gallic acid and ellagic acid; 250 to 100,000 ng/mL for punicalagin). Both samples and standards were analyzed (10 mL injections) in negative ion mode (spray voltage 2.5 kV, ion transfer tube temperature 325 °C, vaporizer temperature 350 °C, sheath gas 50 a.u., aux gas 10 a.u., sweep gas 1 a.u.) using a Q1 resolution of 0.7 *m*/*z* and a Q3 resolution of 1.2 *m*/*z*. The following multiple reaction monitoring (MRM) transitions and collision energies were used: 168.9→79.1 (gallic acid quantifier, CE 23.6), 168.9→125.1 (gallic acid qualifier, CE 14.7), 300.9→145.1 (ellagic acid quantifier, CE 38.4), 300.9→283.9 (ellagic acid qualifier, CE 29.2), 541.1→275.1 (punicalagin quantifier, CE 25.9) and 541.1→301.0 (punicalagin qualifier, CE 27.5). 

Peak integration and quantification were performed in Skyline [32]. Individual standard curves for each analyte were constructed using peak areas from the quantifier transitions. The concentrations of each analyte in the study samples were calculated in an identical manner relative to the regression line. Calibration curves had R2 values ranging from 0.9936 to 0.9997 with a weighting of 1/(x*x). The peak area of the qualifier transition was compared with the peak area of the quantifier transition to generate an ion ratio for compound validation. The comparison of the ion ratios for the standards to the ion ratio of the unknowns (study samples) was carried out with a threshold of 30%.

### 2.6. Statistical Analyses

The error bars in the bar graphs represent the ± standard deviation of the mean. Differences between the control and experimental groups were tested by Student’s *t*-test with significance set at a *p* value ≤ 0.05. Gene expression levels were calculated based on the normalized transcripts per million, and differential expression analysis was performed with the DESeq2 package within Geneious [31]. Genes were considered differentially expressed when they had a Log2 ratio ≤ −2 or ≥2 and a *p* value ≤ 0.05.

## 3. Results

### 3.1. Growth of Lactobacilli in the Presence of Pomegranate Extract

**Figure 1:**
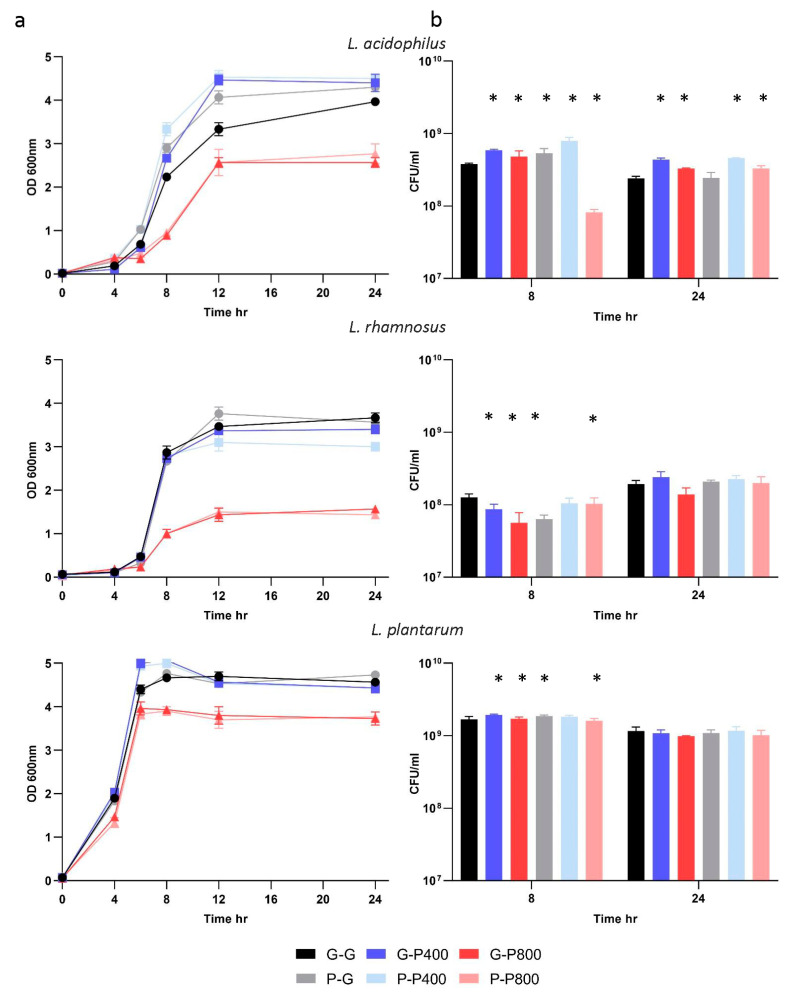
Growth assays of three probiotic strains in the presence of POM extract. (**a**) OD 600 nm values for *L. acidophilus*, *L. rhamnosus* and *L. plantarum* over 24 h at 37 °C. (**b**) CFU/mL counts for each strain at 8 and 24 h. Error bars show the standard deviation of the mean, and * indicates a *p* value ≤ 0.05 when compared to the control G-G. G-G: transfer in SDM with no POM extract and growth in SDM with no POM extract; G-P400: transfer in SDM with no POM extract and growth in SDM with POM 400 µg/mL extract; G-P800: transfer in SDM with no POM extract and growth in SDM with POM 800 µg/mL extract; P-G: transfer in SDM with POM 400 µg/mL extract and growth in SDM with no POM extract; P-P400: transfer in SDM with POM 400 µg/mL extract and growth in SDM with POM 400 µg/mL extract; P-P800: transfer in SDM with POM 400 µg/mL extract and growth in SDM with POM 800 µg/mL extract.

Three commercial probiotic strains were selected for closer evaluation: *L. acidophilus* NCFM (Lac), *L. rhamnosus* GG (Lrh) and *L. plantarum* Lp-115 (Lpl). These strains all grew well in the presence of POM in our initial screen (Figure S1 and Table S1). Preliminary growth assays showed that pomegranate (POM) extract did not support the growth of lactobacilli strains in the absence of an additional sugar source. Therefore, we used a semi-defined medium (SDM) with 0.5% glucose for growth assays. Growth curves over a 24 h period showed that *L. acidophilus* reached the highest OD 600 nm value after growth in P400, whereas the growth profiles were similar for *L. rhamnosus* and *L. plantarum* when grown with and without P400 (Figure 1A). In contrast, OD values were lower for all three strains after 6 h when grown in the higher concentration of P800 (Figure 1A), indicating an inhibitory effect on growth with P800. In the case of *L. acidophilus*, after two transfers in media with P400 and subsequent growth in the control media (P-G), the OD values were higher than those of the control (G-G) (Figure 1A), suggesting that preadaptation to POM in the transfer medium provided, while relatively minor, a growth advantage in the medium without POM (P-G). Next, we enumerated cell counts at T8 and T24 h. These data indicated an increase in colony-forming units of 55% and 81% after 8 and 24 h, respectively, for *L. acidophilus* when grown in P400 compared to the control (G-G) (Figure 1B). For *L. acidophilus*, when the cells were preadapted with two transfers in media containing P400, there was an increase in CFU/mL of 34% at eight hours compared to G-P400. We note that these percent increases in CFU correspond to relatively minor increases in relation to the total viable cell count. Neither *L. rhamnosus* nor *L. plantarum* showed significant differences in cell counts at 24 h across all conditions tested. We performed all subsequent experiments with a concentration of P400, given that these results indicated potential strain inhibition in growth curves with P800.

### 3.2. Flow Cytometry Analysis of Lactobacilli after Growth in Pomegranate

**Figure 2:**
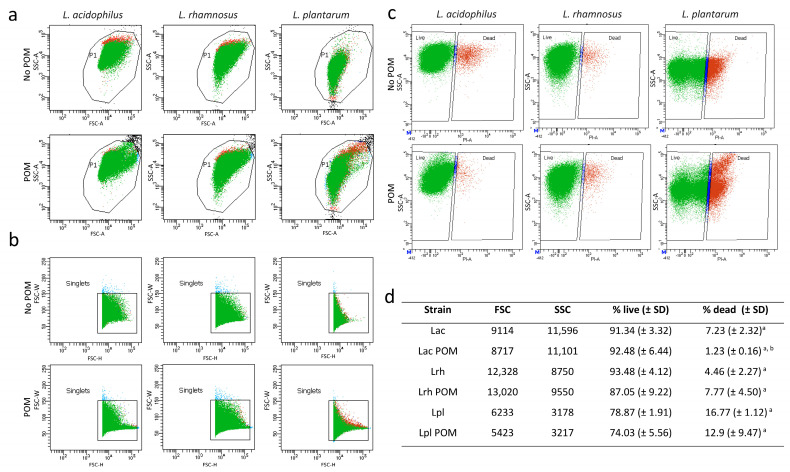
Flow cytometry analysis of probiotic strains grown in the presence of POM extract. Side (**a**) and forward (**b**) plots for each of the three strains. LIVE/DEAD staining with propidium iodide (**c**,**d**). Representative plots with corresponding forward scatter (FSC) and side scatter (SSC) values are shown. In panel (**d**), superscript a denotes a *p* value of ≤ 0.05 when comparing % live and % dead cells, and superscript b denotes a *p* value ≤ 0.05 when comparing % live or % dead values for each strain after growth in POM versus the control with no POM.

We next used flow cytometry to determine the effect of POM in the growth medium for the three strains compared to the control medium with no POM on cell viability, granularity and size. The side scatter and forward scatter plots were similar after growth in POM for *L. acidophilus* and *L. rhamnosus* (Figure 2a,b, respectively), indicating no effect on the cell size or granularity of these strains when POM was present in the growth medium. However, the scatter plots for *L. plantarum* were different between growth conditions, indicating that for this probiotic, POM altered the cell granularity. Using LIVE/DEAD staining, we determined there were significantly fewer dead cells when *L. acidophilus* was grown in P400 compared to the control with no POM (1.23% versus 7.23%, Figure 2c,d). This was not observed with the other two strains. Under both conditions tested, *L. acidophilus* had the lowest number of dead cells, followed by *L. rhamnosus* (4.46% and 7.77%), while close to one-fifth of *L. plantarum* cells were dead (16.77% and 20.9%, Figure 2d). For *L. plantarum*, we observed a higher percentage of dead cells in the presence of POM (20.9%) compared to the control (16.77%); however, this difference was not statistically different (*p* value = 0.07).

### 3.3. Whole-Transcriptome Response of Lactobacilli to Pomegranate

**Figure 3:**
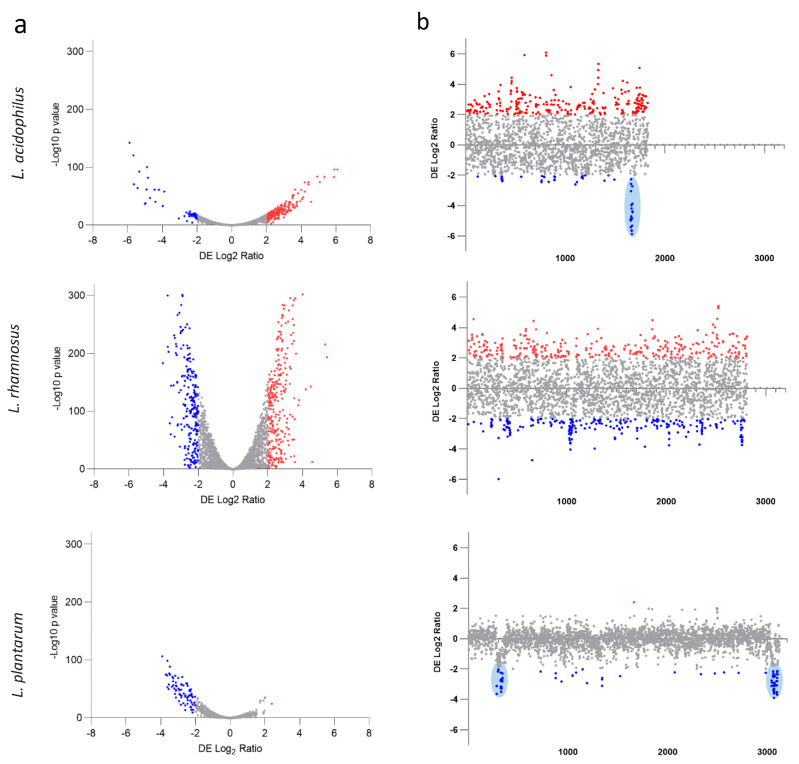
Whole-transcriptome response of probiotic strains to POM extract. Volcano plots comparing Log2 fold change to −Log 10 statistical significance (**a**). Differential gene expression across the chromosome for each of the three strains (**b**). Colors indicate genes with a Log2 fold change ≥ 2 in red and ≤−2 in blue with a *p* value ≤ 0.05. Blue ovals indicate regions discussed in the text.

Given the phenotypic differences after growth with POM extract on the three probiotic strains of interest, we investigated the whole-transcriptome response for each strain to POM extract. As noted with the phenotypes above, the whole-transcriptome response was different for each strain, with 15.88% of the *L. acidophilus* transcriptome, 19.32% of the *L. rhamnosus* transcriptome and only 2.37% of the *L. plantarum* transcriptome differentially expressed (Figure 3a). In addition, of the differentially expressed genes, 240 were upregulated and 51 were downregulated for *L. acidophilus*, 305 were upregulated and 238 were downregulated for *L. rhamnosus*, and only 2 were upregulated and 72 were downregulated for *L. plantarum* (Figure 3a,b and Table S2). Next, the significantly differentially expressed genes were mapped to their chromosomal locations to determine whether there were hotspots of differential gene expression (Figure 3b). These data clearly indicated a region of clustered downregulated genes for *L. acidophilus*, including an ABC transporter gene, a transcriptional regulator gene and a bacteriocin accessory protein-encoding gene (Figure 3b, blue oval). In the case of *L. plantarum*, two notable hotspots of downregulated genes were identified that both include numerous prophage-related genes (Figure 3B, blue ovals), indicating that exposure to POM extract resulted in the potential induction of these phages. 

**Figure 4:**
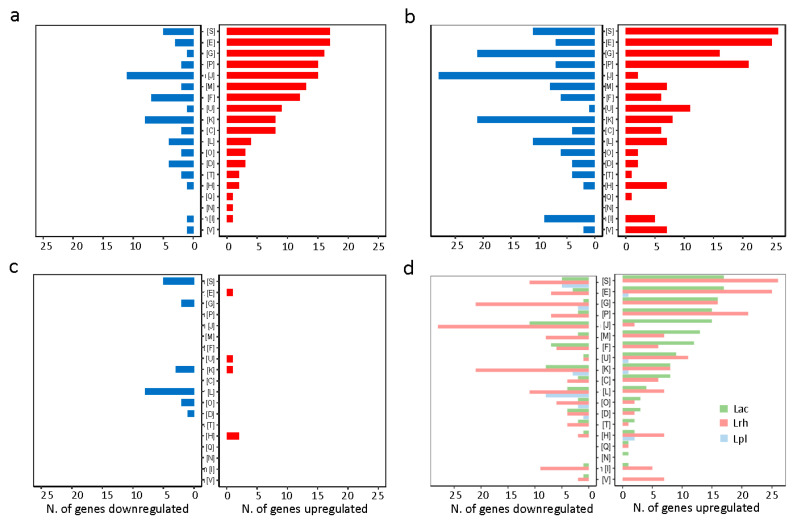
Determination of the clusters of orthologous groups (COG) grouping. COG grouping was assigned to significant genes (Log2 fold change ≥ 2 in red and ≤−2 in blue with a *p* value ≤ 0.05) using the EggNOG database for each strain, (**a**) *L. acidophilus*, Lac, (**b**) *L. rhamnosus*, Lrh, and (**c**) *L. plantarum*, Lpl, and (**d**) an overview of data from the three strains. The COG categories are function unknown [S], amino acid metabolism and transport [E], carbohydrate metabolism and transport [G], inorganic ion transport and metabolism [P], translation [J], cell wall/membrane/envelope biogenesis [M], nucleotide metabolism and transport [F], intracellular trafficking and secretion [U], transcription [K], energy production and conversion [C], replication and repair [L], post-translational modification [O], cell cycle control and mitosis [D], signal transduction mechanisms [T], coenzyme metabolism [H], secondary metabolites biosynthesis [Q], cell motility [N], lipid metabolism [I] and defense mechanism [V].

Subsequently, we used the differentially expressed gene sets to determine the clusters of orthologous groups (COG) grouping for each strain (Figure 4). The COG distribution was different amongst the three strains. For *L. acidophilus* (Figure 4a) and *L. rhamnosus* (Figure 4b) strains, the function unknown [S], amino acid metabolism and transport [E], carbohydrate metabolism and transport [G] and inorganic ion transport and metabolism [P] COGs contained a larger representation of genes, whereas, as expected given the low number of differentially expressed genes for *L. plantarum*, there was less representation across the COGs (Figure 4c,d).

### 3.4. Individual Differential Gene Expression after Growth in POM Extract

We next looked at individual genes of interest that were differentially expressed in each strain after exposure to POM extract. In the case of *L. acidophilus*, differential gene expression ranged from Log2 ± six-fold, with numerous hypothetical proteins amongst the genes with the highest differential gene expression (Table S2). Genes with higher expression in SDM with POM extract compared to the control included a di-tripeptide transport protein (*dtpT*, Log2 ratio 2.99) and genes encoding for multidrug transporter proteins (Log2 ratio 3.74 and 3.36), permeases (Log2 ratio 3.49 and 3.14) and a glycosidase (Log2 ratio 4.12) (Table S2). Interestingly, with the exception of one permease (lba0753), none of these genes were reported as differentially expressed in a previous study from our group, in which the ability of *L. acidophilus* NCFM to metabolize the dietary plant glucosides amygdalin, esculin and salicin was studied [33]. In the case of lba0753, it was found to be upregulated (Log2 ratio 2.3) when grown on esculin compared to lactose. Similarly, for *L. rhamnosus*, differential gene expression ranged from Log2 ± six-fold, with numerous hypothetical proteins amongst the genes with the highest differential gene expression (Table S2). The most upregulated genes included three adjacent genes, ecdB (Log2 ratio 5.41), a putative UbiX-like flavin prenyltransferase gene; *bsdC* (Log2 ratio 5.32), a phenolic acid decarboxylase subunit C gene; and a hypothetical protein (Log2 ratio 5.27). Similar to *L. acidophilus*, a *dtpT* gene was upregulated (Log2 ratio 5.32). Numerous transporter proteins, including those annotated as riboflavin transporters, were also amongst the most upregulated genes, in addition to permeases (Table S2). In the case of *L. plantarum*, only two genes were upregulated, and the expression levels were lower than in the other two strains, with the gene with the highest expression encoding for a transcriptional regulator (*padR*, Log2 ratio 2.39). Other induced genes included, similar to *L. acidophilus* and *L. rhamnosus*, a *dtpT* protein-encoding gene (Log2 ratio 1.97) and a permease gene (Log2 ratio 1.94). Riboflavin synthase (Log2 ratio 1.92) was also one of the seven upregulated genes. As reported above, large proportions of the downregulated genes were part of two separate prophages in the genome of *L. plantarum* (Table S2 and Figure 3D).

### 3.5. Target Metabolite Analysis

**Figure 5:**
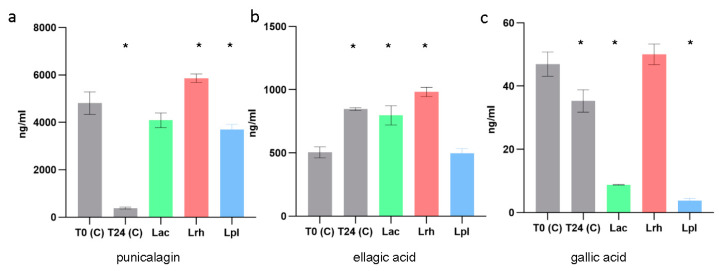
Targeted metabolite analysis for probiotic strains grown with POM extract 400 µg/mL. The ng/mL amount for each metabolite is shown on the y-axis, and the condition is on the x-axis. Punicalagin (**a**), ellagic acid (**b**) and gallic acid (**c**). Gray bars show the control conditions without bacteria. T0 (C): SDM control medium with P400 at 0 h; T24 (C): SDM control medium with P400 at 24 h; Lac: *L. acidophilus*; Lrh: *L. rhamnosus*; and Lpl: *L. plantarum*. Error bars show standard deviation of the mean, and * indicates a *p* value ≤ 0.05 when compared to the control T0.

While lactobacilli are generally considered to lack the required enzymes to bio-transform the main constituents of POM, we measured select biologically relevant metabolites to determine their concentrations after 24 h of growth of the three probiotic strains. Using UPLC-MS/MS, we determined the concentrations of punicalagin, ellagic acid and gallic acid (Figure 5), while urolithins A, C and D were undetected (data not shown). Of the three metabolites, punicalagin was detected in the highest amount (Figure 5a). However, we noted a significant decrease in detection in the control sample after 24 h at 37 °C. This could be due to chemical instability and the degradation of the extract under these conditions. Remarkably, when all three probiotic strains were present, the detected levels of punicalagin were more similar to those in the SDM control medium with P400 (control) at T0, indicating that the presence of the probiotic strains in the medium prevented punicalagin degradation via an undetermined method (Figure 5A). The amount of ellagic acid detected significantly increased at T24 in the control medium and samples with *L. acidophilus* and *L. rhamnosus* but not *L. plantarum* (Figure 5B). In the case of the control T24 *L. acidophilus* samples, this increase could be due to the degradation of punicalagin, whereas the reason for the increased detection in the *L. rhamnosus* sample would need further investigation. The detected amount of gallic acid was not significantly different for *L. rhamnosus* but was significantly reduced for the other two probiotic strains and in the control at T24 compared to T0 (Figure 5C). These data point to the limitations of this experiment, as these results could be further investigated to better understand the interaction of the POM extract, its constituents and their chemical–physical properties, such as solubility and stability in relation to time, temperature, pH and the presence of probiotic strains.

## 4. Discussion

Functional foods are the subject of increased research interest in order to determine the diverse attributes of foods beyond basic nutrition, such as their effects on and bio-transformation by constituents of the GIT [34,35]. Pomegranate is one such functional food consumed in various forms (fruit, juice or extract), which has documented health benefits and encompasses a host of beneficial compounds that are further metabolized into bioactive metabolites in the GIT [11,36]. While the benefits of POM have been postulated for centuries, the beneficial constituents have only recently been identified and studied using scientific methods, including clinical trials [11]. In this study, we set out to further understand the relationship and activities of the functional food POM with lactobacilli and, more specifically, commercially available probiotic bacteria. We focused on commercially available strains from three different species, as these strains are well studied, currently sold as probiotic strains for human consumption and ready for use in formulations with functional foods.

In total, 48 strains of lactobacilli were tested across 14 species, and depending on the strain or species, growth in POM extract had varied effects on OD values (Figure S1). These preliminary results also indicated that, for some strains, P800 was inhibitory and/or slowed the growth rate; therefore, we used a concentration of P400 for subsequent experiments. Our results show that the three probiotic strains displayed different responses to POM. For *L. acidophilus*, POM had a positive effect, as cell growth results showed that growth in POM increased cell numbers at eight hours, and this was further enhanced if the cells had been preadapted by transferring them in POM prior to growth assays. These prior transfers in POM extract may have affected bacterial growth by priming gene expression, such as in the induction of a stress response. There is also the possibility that the addition of POM extract provided minor additional nutritional components. While the increase in cell number was statistically significant in these growth conditions for *L. acidophilus,* we note that the overall increase was relatively minor when compared to the total viable count. Flow cytometry analysis also showed a lower amount of cell death when *L. acidophilus* was grown with POM, indicating a protective effect on the cells yet to be established. Given the results we observed with *L. acidophilus* in particular and the lack of previously published studies with similar observations for this species, it would be interesting for future studies to further establish any potential mutual benefit. *L. plantarum* and *L. rhamnosus* grew to the same extent as the controls in the presence of POM, and the preadaptation did not result in enhanced growth; this was confirmed by enumerating cell counts. Previous work also determined that the growth of probiotic species was relatively unaffected by the inclusion of POMx in the growth medium [18]. A subsequent study determined that POMx significantly enhanced lactobacilli numbers in the batch culture fermentation of fecal slurry [19]. A third study determined an increase in the cell counts of total lactobacilli with P400; however, the individual species’ details are not provided [20]. 

We next grew all three strains in the presence of POM extract and determined the whole-transcriptome response during the logarithmic phase. Strikingly, we determined three different global responses amongst the three strains. Exposure to POM extract had the most significant and highest level of whole-transcriptome response for *L. rhamnosus*, followed by *L. acidophilus*, where the upregulation of the transcriptome was more impacted. The least responsive transcriptome to POM extract was *L. plantarum*’s, with less than three percent of the genome impacted and few upregulated genes. Both *L. plantarum* and *L. rhamnosus* have significantly larger genomes (~3.2 and 3 Mb, respectively) than *L. acidophilus* (~2 Mb) and are therefore presumed to be less fastidious. In particular, since many *L. plantarum* strains are derived from plants and fermented foods [37], it is plausible that this species has evolved with plant products and thus has a distinct transcriptional response. Genes encoding for numerous types of transporters, such as phosphotransferase (PTS) system, multidrug, di-tripeptide transporters and members of the major facilitator superfamily (MFS) transporter families, were upregulated. In addition, genes encoding for glucosidases, permeases, hydrolases and hypothetical proteins were upregulated. While all three strains showed a markedly different global transcriptional response, there was some commonality; in particular, homologs of certain genes were upregulated, for example, *dtpT*, a di-tripeptide transporter, strongly indicating their involvement in the exposure and/or growth of these strains in the presence of POM extract. Further, in silico analysis of these genes of interest and the construction of strains devoid of these genes could be performed to determine the role of these genes in the phenotypes described in this study. For example, future studies should determine whether the expression of these genes contributes to the lower number of dead cells determined by flow cytometry for *L. acidophilus* when grown in P400. Likewise, it should be assessed whether induction of prophages from the *L. plantarum* genome occurred and contributed to cell death in the total cell population. 

We also detected specific metabolites derived from POM after growth across the three strains. We included these metabolites, as punicalagin is the precursor to the more bioavailable ellagic acid, and all three have shown beneficial attributes in the gut [11]. For example, punicalagin, ellagic acid and gallic acid demonstrated anti-inflammatory effects [38,39] and pathogen inhibition [40,41,42]. The lack of detection of urolithins was not unexpected, as this correlates with previous reports, including studies with lactobacilli [20,43], and other members of the gut microbiota have been shown to possess the genes required for the conversion to urolithin [44]. While none of the urolithins were detected, we were able to measure the concentrations of punicalagin, ellagic acid and gallic acid. We did note a lack of degradation in the case of punicalagin when compared to the control, indicating a possible protective effect by the probiotic strains. This protective effect could result in the greater bioavailability of punicalagin to other members of the microbiome that are known to metabolize punicalagin to urolithins [44]. However, these preliminary data reveal that additional studies are needed to determine the interaction of the POM extract and its constituents with probiotic strains. Future considerations should include their chemical–physical properties, such as solubility and stability in relation to time and temperature. 

## 5. Conclusions

Overall, this study investigates the interplay between a beneficial dietary compound and commercially relevant probiotic bacteria, shows that POM extract triggers species-specific responses by probiotic strains and substantiates the rising interest in using POM as a prebiotic compound. The data presented here point to the need for continued investigation to further discover the constituents of POM that result in the phenotypes we have observed. Especially intriguing is how a functional food such as POM can discern and act to inhibit pathogenic members of the GIT while leaving beneficial bacteria such as *Lactobacillaceae* species unscathed or even enhanced in number. We newly report on the transcriptome response of the three strains, emphasizing the results we have determined throughout: POM extract triggers species-specific responses by probiotic strains. Also compelling is the lack of punicalagin degradation in the media containing the probiotic strains. Overall, this study suggests an under-appreciated evolutionary complexity between functional foods and/or their metabolites and the bacteria of the GIT, and opens new avenues to include POM in probiotic formulations and expand our understanding of how select dietary compounds impact the physiology of beneficial bacteria and ultimately the health of the host.

## 6. Patents

S.O.F. and R.B. are inventors on patent 17/442,776: Probiotic bacteria capable of adaptive response to pomegranate extract and methods of production and use thereof.

## Data Availability

The RNA-seq data were deposited in the NCBI Short Read Archive (SRA) database under the BioProject ID PRJNA907268. The SRA accessions number are SAMN31967636–SAMN31967647.

## Supplementary Materials

The following supporting information can be downloaded at https://www.mdpi.com/article/10.3390/microorganisms11020404/s1. Figure S1: Schematic of growth conditions; Figure S2: OD values of strains grown with POM extract; Table S1: Details of strains used in Figure S2; Table S2: List of differentially expressed genes from whole-transcriptome analysis.
