# Supplementary material for: Impact of Pomegranate on Probiotic Growth, Viability, Transcriptome and Metabolism
Source: Microorganisms. 2023 Feb 5;11(2):404. doi: 10.3390/microorganisms11020404 (PMC9964784; doi:10.3390/microorganisms11020404)
Supplement: Supplementary file 1 [file microorganisms-11-00404-s001.zip › microorganisms-2133659-supplementary.pdf]

O’Flaherty et al 2133659

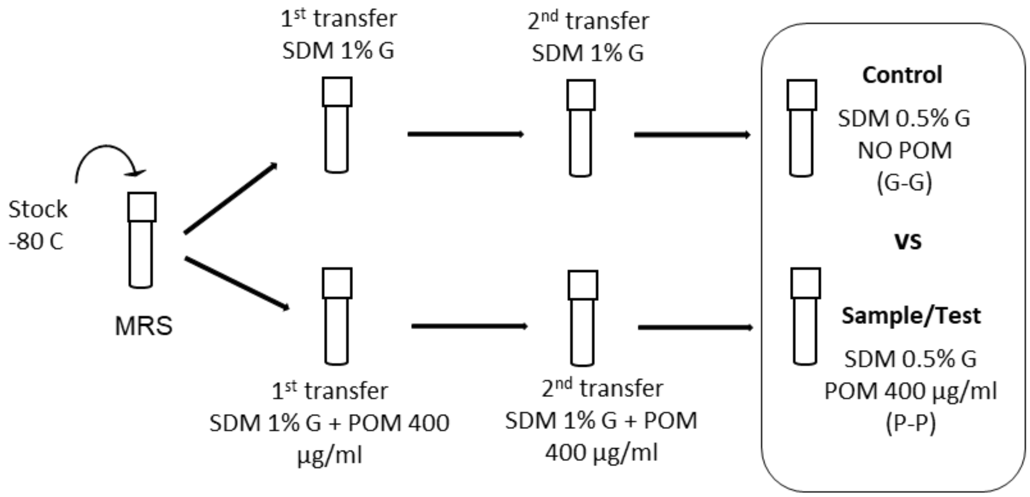

Figure S1

Schematic of experimental design for experiments

Materials and Methods for Figure S2:

Strains (Table S1) were inoculated (1%) from -80°C glycerol stocks into MRS broth and grown over night at 37°C under ambient atmospheric conditions. These cultures were then used to inoculate (1%) SDM medium (1% glucose, with or without POM 400 µg/ml) for two overnight transfers at 37°C prior to growth assays. Growth assays were performed in 96 microtiter well plates in SDM (0.5% glucose, control) and SDM (0.5% glucose with 400 or 800 µg/ml POM extract). The OD 600nm was recorded every hour over 24 hours using a microtiter plate reader (BMG Technologies). Ninety six well growth assays were repeated at least twice with triplicate technical replicates for each sample.

SDM Composition:

Kimmel, S.A.; Roberts, R.F. Development of a growth medium suitable for exopolysaccharide production by Lactobacillus delbrueckii ssp. bulgaricus RR. *Int J Food Microbiol* **1998**, *40*, 87-92, doi:10.1016/s0168-1605(98)00023-3.

The following components were weighed out and dissolved in 1L of water to make SDM medium (without glucose): 1g tween 80, 2g ammonium citrate, 5g sodium acetate, 0.1g magnesium sulfate heptahydrate, 0.05g Manganese (II) sulfate, 2g dipotassium phosphate, 5g yeast nitrogen base and 10g bacto casitone. Once dissolved the SDM was autoclaved at 121°C for 20 minutes.

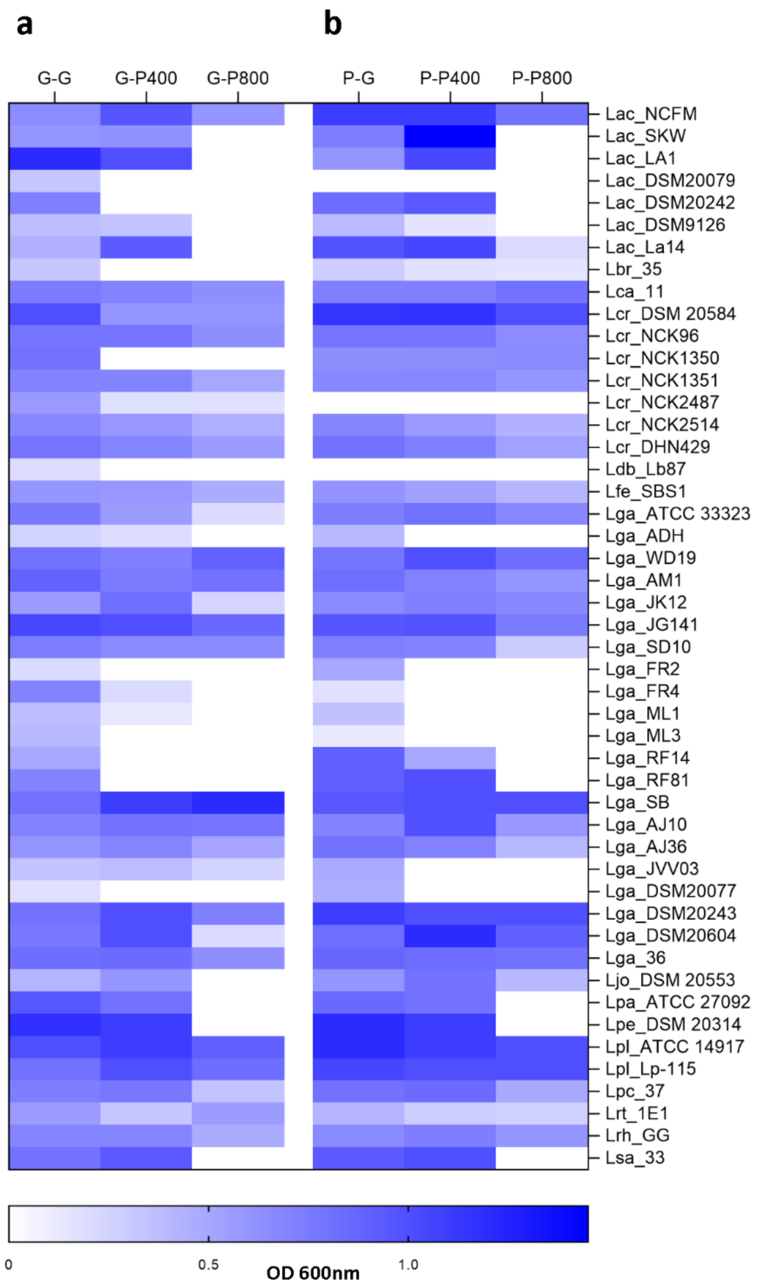

Figure S2. OD 600 nm values for strains transferred twice in glucose (a, control) or transferred twice in media with POM 400 µg/ml extract (b). In panel a, G-G; transfer in SDM no POM extract and growth in SDM no POM extract, G-P400; transfer in SDM no POM extract and growth in SDM with POM 400 µg/ml extract, G-P800; transfer in SDM no POM extract and growth in SDM with POM 800 µg/ml extract. In panel b, P-G; transfer in SDM with POM 400 µg/ml extract and growth in SDM no POM extract, P-P400; transfer in SDM with POM 400 µg/ml extract and growth in SDM with POM 400 µg/ml extract, P-P800; transfer in SDM with POM 400 µg/ml extract and growth in SDM with POM 800 µg/ml extract. Strain details are provided in Table S1.

Table S1: Table of strains used in Figure S1.

Species		Strain	Strain Code
Lactobacillus	*acidophilus*	NCFM	Lac_NCFM
Lactobacillus	*acidophilus*	SKW	Lac_SKW
Lactobacillus	*acidophilus*	LA-1	Lac_LA1
Lactobacillus	*acidophilus*	DSM-20079	Lac_DSM20079
Lactobacillus	*acidophilus*	DSM-20242	Lac_DSM20242
Lactobacillus	*acidophilus*	DSM-9126	Lac_DSM9126
Lactobacillus	*acidophilus*	La-14	Lac_La14
Levilactobacillus	*brevis*	Lbr-35	Lbr_35
Lacticaseibacillus	*casei*	Lc11	Lca_11
Lactobacillus	*crispatus*	DSM 20584	Lcr_DSM 20584
Lactobacillus	*crispatus*	NCK96	Lcr_NCK96
Lactobacillus	*crispatus*	NCK1350	Lcr_NCK1350
Lactobacillus	*crispatus*	NCK1351	Lcr_NCK1351
Lactobacillus	*crispatus*	DSM-20356	Lcr_NCK2487
Lactobacillus	*crispatus*	JV-V01	Lcr_NCK2514
Lactobacillus	*crispatus*	DHN-429	Lcr_DHN429
Lactobacillus	*delbreckii subsp.bulgaricus*	Lb-87	Ldb_Lb87
Limosilactobacillus	*fermentum*	SBS-1	Lfe_SBS1
Lactobacillus	*gasseri*	ATCC 33323	Lga_ATCC 33323
Lactobacillus	*gasseri*	ADH	Lga_ADH
Lactobacillus	*gasseri*	WD19	Lga_WD19
Lactobacillus	*gasseri*	AM1	Lga_AM1
Lactobacillus	*gasseri*	JK12	Lga_JK12
Lactobacillus	*gasseri*	JG141	Lga_JG141

Lactobacillus	*gasseri*	SD10	Lga_SD10
Lactobacillus	*gasseri*	FR2	Lga_FR2
Lactobacillus	*gasseri*	FR4	Lga_FR4
Lactobacillus	*gasseri*	ML1	Lga_ML1
Lactobacillus	*gasseri*	ML3	Lga_ML3
Lactobacillus	*gasseri*	RF14	Lga_RF14
Lactobacillus	*gasseri*	RF81	Lga_RF81
Lactobacillus	*gasseri*	S-B	Lga_SB
Lactobacillus	*gasseri*	AJ 10	Lga_AJ10
Lactobacillus	*gasseri*	AJ 36	Lga_AJ36
Lactobacillus	*gasseri*	JV-V03	Lga_JVV03
Lactobacillus	*gasseri*	DSM-20077	Lga_DSM20077
Lactobacillus	*gasseri*	DSM-20243	Lga_DSM20243
Lactobacillus	*gasseri*	DSM-20604	Lga_DSM20604
Lactobacillus	*gasseri*	La-36	Lga_36
Lactobacillus	*gasseri*	DSM 20553	Ljo_DSM 20553
Lactobacillus	*johnsonii*	ATCC 27092	Lpa_ATCC 27092
Lacticaseibacillus	*paracasei*	DSM 20314	Lpe_DSM 20314
Lactiplantibacillus	*pentosus*	ATCC 14917	Lpl_ATCC 14917
Lactiplantibacillus	*plantarum*	Lp-115	Lpl_Lp-115
Lactiplantibacillus	*plantarum*	Lpc-37	Lpc_37
Lactiplantibacillus	*plantarum*	1E1	Lrt_1E1
Lacticaseibacillus	*rhamnosus*	GG (ATCC 53103)	Lrh_GG
Ligilactobacillus	*salivarius*	Ls-33	Lsa_33

Table S2 List of differentially expressed genes from whole transcriptomic analysis

	**Lactobacillus acidophilus NCFM**		
	**Name**	**Differential Expression Log2 Ratio**	**Differential Expression p-value**
1	hypothetical protein CDS	6.09	2.56E-99
2	hypothetical protein CDS	5.92	1.38E-99
3	hypothetical protein CDS	5.89	3.34E-86
4	glpF CDS	5.34	4.77E-86
5	NLP-P60 family secreted protein CDS	5.08	2.63E-77
6	hypothetical protein CDS	4.94	1.21E-86
7	hypothetical protein CDS	4.59	1.16E-42
8	aggregation promoting protein CDS	4.42	1.73E-73
9	dihydroxyacetone kinase CDS	4.42	3.36E-77
10	exoA CDS	4.21	1.34E-76
11	hypothetical protein CDS	4.21	1.15E-60
12	glycosidase CDS	4.12	5.89E-51
13	celB CDS	4.06	1.32E-47
14	dihydroxyacetone kinase subunit DhaK CDS	4.01	1.77E-63
15	hypothetical protein CDS	3.95	6.61E-44
16	macrolide efflux protein CDS	3.81	1.40E-57
17	permease CDS	3.77	5.32E-43
18	lysM CDS	3.76	1.50E-35
19	multidrug transporter CDS	3.74	8.14E-40
20	glpF CDS	3.73	3.28E-35
21	peroxidase (Npx) CDS	3.66	3.20E-64
22	PTS system transporter subunit IIA CDS	3.63	2.21E-33
23	hypothetical protein CDS	3.63	1.46E-31
24	hypothetical protein CDS	3.57	9.82E-55
25	hypothetical protein CDS	3.55	1.09E-37
26	hypothetical protein CDS	3.54	2.98E-52
27	acetyltransferase CDS	3.54	1.10E-53
28	hypothetical protein CDS	3.53	3.38E-33
29	hypothetical protein CDS	3.51	3.45E-54
30	transposase CDS	3.49	1.21E-49
31	hydrolase CDS	3.49	1.79E-37
32	lmrB CDS	3.42	2.75E-27
33	hypothetical protein CDS	3.40	4.60E-28
34	malH CDS	3.39	1.76E-42
35	permease CDS	3.37	4.44E-41
36	repB CDS	3.36	1.16E-42
37	hypothetical protein CDS	3.36	1.38E-40
38	hypothetical protein CDS	3.36	1.52E-26

39	hypothetical protein CDS	3.32	4.34E-38
40	lysA CDS	3.30	8.93E-42
41	channel-forming protein CDS	3.30	8.03E-32
42	amino acid permease CDS	3.29	1.64E-35
43	hypothetical protein CDS	3.28	6.50E-28
44	ackA CDS	3.27	2.94E-28
45	Na-H antiporter CDS	3.23	2.29E-26
46	hypothetical protein CDS	3.23	8.10E-34
47	xanthine-uracil permeases CDS	3.22	5.83E-24
48	multi-drug-type permease CDS	3.14	2.68E-44
49	hypothetical protein CDS	3.12	2.10E-24
50	hypothetical protein CDS	3.11	9.89E-29
51	maltose ABC transporter permease CDS	3.10	1.74E-20
52	50S ribosomal protein L33 CDS	3.09	1.87E-37
53	hypothetical protein CDS	3.08	1.39E-40
54	potassium uptake protein Kup CDS	3.08	8.16E-34
55	PTS system arbutin-like transporter subunit IIBC CDS	3.07	1.98E-28
56	yifK CDS	3.04	1.79E-35
57	aapA CDS	3.03	1.86E-34
58	multidrug transporter CDS	3.02	4.17E-27
59	nicotinamide mononucleotide CDS	3.01	1.44E-20
60	hypothetical protein CDS	3.00	6.71E-15
61	secE CDS	3.00	1.57E-29
62	dtpT CDS	2.98	1.33E-29
63	hypothetical protein CDS	2.95	2.37E-27
64	cation transporter CDS	2.94	2.87E-25
65	polysaccharide transporter CDS	2.94	1.21E-27
66	hypothetical protein CDS	2.94	6.22E-26
67	hypothetical protein CDS	2.93	2.13E-30
68	maltose ABC transporter permease CDS	2.92	6.99E-23
69	hypothetical protein CDS	2.90	3.14E-21
70	pyrF CDS	2.90	4.78E-30
71	membrane protein CDS	2.89	9.48E-22
72	permease CDS	2.89	2.12E-37
73	site-specific recombinase CDS	2.88	2.82E-30
74	clpP CDS	2.88	7.20E-35
75	hypothetical protein CDS	2.87	2.01E-34
76	UDP-N-acetylglucosamine 1-carboxyvinyltransferase	2.84	1.86E-28
77	pyrE CDS	2.84	5.69E-30
78	gntR CDS	2.83	1.27E-29
79	ssdh CDS	2.81	1.58E-36
80	hypothetical protein CDS	2.80	5.04E-26
81	transmembrane protein CDS	2.79	2.92E-25

82	hypothetical protein CDS	2.79	2.19E-26
83	receptor CDS	2.78	4.18E-24
84	hypothetical protein CDS	2.78	7.69E-29
85	rpsP CDS	2.78	1.22E-23
86	poxB CDS	2.77	5.53E-32
87	signal peptidase I CDS	2.77	5.80E-30
88	hypothetical protein CDS	2.77	4.01E-31
89	acyltransferase CDS	2.77	5.43E-30
90	site-specific recombinase CDS	2.74	5.31E-29
91	cell wall-associated hydrolase CDS	2.73	1.98E-29
92	hypothetical protein CDS	2.72	9.77E-27
93	amino acid permease CDS	2.72	6.98E-20
94	hypothetical protein CDS	2.72	6.06E-24
95	cloride channel-like protein CDS	2.72	3.16E-30
96	neopullulanase CDS	2.72	4.92E-23
97	4-methyl-5(beta-hydroxyethyl)-thiazole monophosphate synthesis	2.71	9.43E-27
98	multidrug resistance protein CDS	2.71	1.44E-14
99	uracil permease CDS	2.71	7.91E-18
100	multidrug resistance protein CDS	2.70	1.28E-13
101	guanylate kinase CDS	2.69	9.86E-30
102	hypothetical protein CDS	2.69	1.79E-12
103	amino acid permease CDS	2.69	3.92E-30
104	sugar ABC transporter permease CDS	2.69	5.09E-31
105	30S ribosomal protein S15 CDS	2.69	1.88E-21
106	uspA CDS	2.68	2.09E-23
107	hypothetical protein CDS	2.68	1.43E-12
108	hypothetical protein CDS	2.67	1.14E-22
109	transporter protein CDS	2.67	4.07E-19
110	oxidoreductase CDS	2.67	1.06E-28
111	hypothetical protein CDS	2.67	3.20E-22
112	gadC CDS	2.66	1.46E-23
113	PTS system cellobiose-specific transporter subunit IIC CDS	2.66	8.69E-20
114	cation-transporting ATPase CDS	2.65	1.15E-33
115	rpmF CDS	2.65	5.48E-20
116	ribosomal-protein-alanine N-acetyltransferase CDS	2.65	7.01E-24
117	rodA CDS	2.64	4.25E-24
118	rpmE2 CDS	2.63	1.72E-19
119	Na+-transporting ATP synthase CDS	2.63	1.85E-23
120	NAD+ binding protein for K+transport CDS	2.62	2.67E-26
121	hypothetical protein CDS	2.61	6.58E-23
122	portal protein CDS	2.61	1.06E-24
123	prophage DNA packaging protein NU1 CDS	2.60	5.01E-14
124	aprT CDS	2.60	3.60E-22

125	acetyltransferase CDS	2.59	4.95E-24
126	amino acid permease CDS	2.58	5.52E-25
127	rplJ CDS	2.58	1.31E-28
128	hypothetical protein CDS	2.56	5.57E-14
129	hypothetical protein CDS	2.54	1.79E-22
130	rplS CDS	2.53	2.98E-19
131	undecaprenyl-phosphaten-acetyl- glucosaminyltransfera se CDS	2.53	8.23E-31
132	hypothetical protein CDS	2.53	2.27E-24
133	hypothetical protein CDS	2.53	7.25E-22
134	acetyltransferase CDS	2.52	1.58E-29
135	hypothetical protein CDS	2.52	2.00E-12
136	pgmB CDS	2.51	1.86E-18
137	hypothetical protein CDS	2.50	1.24E-18
138	aa transporter CDS	2.49	2.47E-23
139	hypothetical protein CDS	2.48	6.43E-12
140	hypothetical protein CDS	2.48	1.00E-23
141	rplU CDS	2.48	1.37E-22
142	xanthine-uracil permeases family protein CDS	2.47	6.73E-16
143	mucus binding protein CDS	2.47	3.81E-18
144	Na+-H+ antiporter CDS	2.46	1.21E-21
145	rbsD CDS	2.46	3.79E-11
146	K+ uptake protein CDS	2.46	1.07E-22
147	low temperature requirement A protein CDS	2.46	7.85E-22
148	prsA CDS	2.43	1.45E-18
149	hypothetical protein CDS	2.43	2.27E-18
150	thiD CDS	2.42	1.31E-27
151	amino acid permease CDS	2.42	3.75E-21
152	hypothetical protein CDS	2.42	4.91E-26
153	multidrug resistance efflux pump CDS	2.39	1.83E-17
154	fibrinogen-binding protein CDS	2.39	2.99E-06
155	rpmA CDS	2.39	1.85E-19
156	glutamine amidotransferase CDS	2.39	6.88E-24
157	aa transporter CDS	2.38	7.35E-18
158	sugar ABC transporter CDS	2.37	1.28E-24
159	hypothetical protein CDS	2.37	7.07E-23
160	hypothetical protein CDS	2.36	2.08E-21
161	amino acid permease CDS	2.36	1.69E-18
162	rpsU CDS	2.35	4.55E-16
163	site-specific recombinase CDS	2.34	6.62E-21
164	ldhD CDS	2.31	5.12E-21
165	permease CDS	2.31	4.03E-26
166	sugar kinase CDS	2.31	5.92E-20
167	bshB CDS	2.30	1.56E-21

168	pyrD CDS	2.30	3.92E-22
169	hypothetical protein CDS	2.29	3.56E-15
170	branched-chain amino acid transporter CDS	2.29	3.18E-14
171	hypothetical protein CDS	2.29	8.05E-15
172	repB CDS	2.29	2.27E-16
173	hypothetical protein CDS	2.28	0.00101667
174	hypothetical protein CDS	2.28	2.80E-12
175	hypothetical protein CDS	2.28	2.29E-17
176	hypothetical protein CDS	2.27	1.13E-16
177	ldh CDS	2.27	5.85E-25
178	phospho-beta-galactosidase I CDS	2.27	5.47E-23
179	rplL CDS	2.26	1.09E-21
180	3-methyladenine DNA glycosylase CDS	2.26	2.52E-19
181	PTS system cellobiose-specific transporter subunit IIC CDS	2.26	2.90E-17
182	fructokinase CDS	2.25	1.03E-15
183	galactose-1-phosphate uridylyltransferase CDS	2.23	3.31E-21
184	oxalyl-CoA decarboxylase CDS	2.23	1.75E-24
185	oligo-1,6-glucosidase CDS	2.23	9.20E-14
186	cadX CDS	2.22	1.26E-20
187	ABC transporter substrate binding protein CDS	2.22	8.91E-16
188	aspartate alanine antiporter CDS	2.21	5.22E-13
189	dedA CDS	2.21	1.27E-19
190	inosine-5'-monophosphate dehydrogenase CDS	2.21	3.97E-19
191	pyrR CDS	2.21	4.04E-20
192	epsV CDS	2.20	1.50E-21
193	hypothetical protein CDS	2.20	4.82E-22
194	hypothetical protein CDS	2.20	6.05E-18
195	transmembrane protein CDS	2.19	1.58E-20
196	hypothetical protein CDS	2.19	2.32E-14
197	hypothetical protein CDS	2.18	1.16E-18
198	hypothetical protein CDS	2.17	1.56E-15
199	crcB CDS	2.17	2.18E-16
200	plnI CDS	2.17	2.94E-17
201	crcB CDS	2.17	4.73E-15
202	oxidoreductase CDS	2.17	4.41E-23
203	hypothetical protein CDS	2.15	4.36E-19
204	celB CDS	2.15	6.10E-10
205	hypothetical protein CDS	2.15	3.04E-09
206	transporter protein CDS	2.14	5.17E-18
207	hypothetical protein CDS	2.14	1.16E-13
208	msrA CDS	2.13	3.51E-21
209	phosphotransferase system enzyme II CDS	2.12	1.31E-10
210	hypothetical protein CDS	2.12	1.03E-14

211	rhodanese-related sulfurtransferase CDS	2.12	3.45E-15
212	protease CDS	2.12	1.30E-14
213	arsC CDS	2.12	1.11E-21
214	hypothetical protein CDS	2.10	3.93E-15
215	pthA CDS	2.10	3.55E-22
216	transcriptional regulator CDS	2.10	1.95E-13
217	amino acid permease CDS	2.10	5.44E-15
218	HAD family hydrolase CDS	2.09	3.90E-08
219	glnP CDS	2.09	9.86E-15
220	phosphoglycerate mutase CDS	2.08	1.86E-18
221	pyrR CDS	2.08	1.63E-10
222	6-phospho-beta-glucosidase CDS	2.08	1.02E-10
223	oppA CDS	2.07	8.68E-06
224	Na+-H+-exchanging protein CDS	2.07	5.35E-11
225	oxidoreductase CDS	2.07	3.79E-06
226	sugar transporter CDS	2.07	1.50E-11
227	hypothetical protein CDS	2.05	2.26E-12
228	secG CDS	2.05	8.55E-12
229	hypothetical protein CDS	2.05	5.30E-16
230	hypothetical protein CDS	2.04	4.93E-17
231	hypothetical protein CDS	2.04	8.24E-06
232	hypothetical protein CDS	2.04	6.02E-16
233	hypothetical protein CDS	2.03	2.40E-16
234	rpmG CDS	2.03	1.63E-13
235	hypothetical protein CDS	2.02	7.92E-18
236	acpD CDS	2.02	4.18E-11
237	mreD CDS	2.02	6.39E-20
238	hypothetical protein CDS	2.00	1.02E-13
239	lacS CDS	2.00	1.35E-11
240	epsU CDS	2.00	8.97E-19
241	hypothetical protein CDS	1.99	2.64E-19
242	hypothetical protein CDS	1.98	7.98E-12
243	licT CDS	1.98	0.00015681
244	rpoZ CDS	1.97	1.04E-15
245	oxidoreductase CDS	1.96	4.33E-19
246	hydrolase CDS	1.96	1.66E-17
247	hypothetical protein CDS	1.95	3.63E-16
248	hypothetical protein CDS	1.94	1.53E-16
249	HAD family hydrolase CDS	1.94	9.81E-06
250	mreB CDS	1.94	4.76E-18
251	hypothetical protein CDS	1.94	8.84E-07
252	hypothetical protein CDS	1.94	3.36E-15
253	hypothetical protein CDS	1.94	1.81E-05

254	hypothetical protein CDS	1.93	7.67E-16
255	cysteine synthase CDS	1.93	4.02E-05
256	PTS system transporter subunit IIBC CDS	1.93	1.84E-13
257	scrA CDS	1.93	2.88E-09
258	oppC CDS	1.93	1.61E-13
259	di-tripeptide transporter CDS	1.93	7.11E-17
260	rpoB CDS	1.92	1.02E-18
261	rpmB CDS	1.92	7.94E-13
262	glycosyltransferase CDS	1.92	1.35E-15
263	ftsW CDS	1.92	1.21E-14
264	tuf CDS	1.92	3.21E-13
265	malate permease CDS	1.92	2.85E-16
266	amino acid permease CDS	1.92	4.60E-15
267	hypothetical protein CDS	1.91	1.68E-12
268	hypothetical protein CDS	1.91	4.70E-12
269	hypothetical protein CDS	1.91	6.61E-15
270	hypothetical protein CDS	1.90	3.97E-06
271	amino acid permease CDS	1.90	7.81E-17
272	hypothetical protein CDS	-1.90	1.90E-11
273	sugar ABC transporter ATP binding protein CDS	-1.91	7.63E-17
274	transcriptional regulator CDS	-1.91	3.64E-12
275	hypothetical protein CDS	-1.91	1.45E-16
276	signal peptidase CDS	-1.91	5.13E-14
277	regulator CDS	-1.92	1.03E-05
278	ATPase CDS	-1.92	1.36E-15
279	mucus binding protein CDS	-1.92	1.75E-10
280	rplX CDS	-1.93	2.88E-15
281	recN CDS	-1.93	1.78E-11
282	infB CDS	-1.93	1.35E-17
283	4-hydroxy-tetrahydrodipicolinate synthase CDS	-1.93	5.41E-12
284	dCMP deaminase CDS	-1.93	2.40E-17
285	hypothetical protein CDS	-1.97	9.63E-20
286	DNA primase CDS	-1.97	7.36E-18
287	transcriptional regulator CDS	-1.97	1.40E-16
288	DNA replication intiation control protein YabA CDS	-1.97	1.13E-09
289	arginase CDS	-1.98	6.48E-17
290	parB CDS	-1.99	4.01E-14
291	purE CDS	-2.00	6.50E-17
292	coaE CDS	-2.01	7.62E-13
293	chromosome segregation protein Smc CDS	-2.02	1.97E-13
294	cbiO CDS	-2.05	3.63E-17
295	hypothetical protein CDS	-2.06	7.44E-16
296	tsf CDS	-2.06	3.39E-20

297	DNA polymerase III subunit delta CDS	-2.06	6.02E-14
298	cbiO CDS	-2.07	1.20E-19
299	xerD CDS	-2.08	4.85E-19
300	tetrapyrrole methylase CDS	-2.08	6.75E-15
301	tetR CDS	-2.09	3.34E-16
302	tig CDS	-2.10	1.36E-17
303	phosphoketolase CDS	-2.11	1.97E-16
304	cytidin monophosphate kinase CDS	-2.12	2.46E-17
305	clpX CDS	-2.13	2.45E-22
306	L7A family ribosomal protein CDS	-2.14	8.28E-19
307	hypothetical protein CDS	-2.14	4.77E-17
308	glycoprotein endopeptidase CDS	-2.15	1.24E-16
309	rpsB CDS	-2.21	2.51E-21
310	hypothetical protein CDS	-2.26	2.82E-19
311	hypothetical protein CDS	-2.27	2.11E-05
312	hypothetical protein CDS	-2.29	3.75E-17
313	fmtB CDS	-2.29	1.41E-18
314	ribT CDS	-2.30	4.21E-18
315	dehydrogenase CDS	-2.31	7.34E-20
316	hypothetical protein CDS	-2.33	1.54E-18
317	ADP-ribose pyrophosphatase CDS	-2.35	2.54E-20
318	miaA CDS	-2.36	5.99E-21
319	2-deoxyribose-5-phosphate aldolase CDS	-2.39	1.26E-25
320	capsid protein CDS	-2.44	4.96E-17
321	hypothetical protein CDS	-2.46	1.14E-20
322	cold-shock protein CDS	-2.46	4.27E-22
323	hypothetical protein CDS	-2.57	2.92E-08
324	gntR CDS	-2.61	1.27E-23
325	hypothetical protein CDS	-2.72	2.73E-17
326	transcriptional regulator CDS	-3.04	5.36E-13
327	hypothetical protein CDS	-3.88	1.50E-60
328	hypothetical protein CDS	-3.97	2.65E-35
329	hypothetical protein CDS	-4.19	5.14E-64
330	hypothetical protein CDS	-4.39	9.09E-43
331	hypothetical protein CDS	-4.44	3.18E-64
332	hypothetical protein CDS	-4.71	1.55E-49
333	plnG CDS	-4.82	7.59E-85
334	response regulator CDS	-4.87	4.94E-104
335	ABC transporter ATP-binding protein CDS	-4.90	1.59E-64
336	hypothetical protein CDS	-4.96	5.36E-40
337	hypothetical protein CDS	-4.99	6.71E-39
338	hypothetical protein CDS	-5.32	1.03E-95
339	hypothetical protein CDS	-5.42	5.34E-67

340	gassericin K7 B accessory protein CDS	-5.64	2.80E-73
341	hypothetical protein CDS	-5.66	1.94E-124
342	histidine kinase CDS	-5.88	2.30E-146
	**Lactobacillus rhamnosus GG**		
	**Name**	**Differential Expression Log2 Ratio**	**Differential Expression p-value**
1	ecdB CDS	5.41	1.07E-195
2	bsdC CDS	5.32	5.50E-218
3	hypothetical protein CDS	5.27	0
4	hypothetical protein CDS	4.56	4.49E-13
5	ribZ 1 CDS	4.56	0
6	putative ABC transporter ATP-binding protein CDS	4.48	1.92E-144
7	hypothetical protein CDS	4.43	0
8	hypothetical protein CDS	4.19	5.29E-140
9	hypothetical protein CDS	4.02	6.78E-305
10	ribU CDS	3.91	0
11	yhdG 1 CDS	3.90	0
12	hypothetical protein CDS	3.89	0
13	hypothetical protein CDS	3.88	0
14	uidB CDS	3.81	8.53E-121
15	hypothetical protein CDS	3.76	5.41E-112
16	mepA CDS	3.65	0
17	pgpB CDS	3.63	9.08E-202
18	ybiR CDS	3.59	2.06E-297
19	hypothetical protein CDS	3.57	9.03E-13
20	hypothetical protein CDS	3.55	5.62E-88
21	hypothetical protein CDS	3.55	0
22	hypothetical protein CDS	3.53	4.29E-124
23	hypothetical protein CDS	3.52	1.25E-23
24	hypothetical protein CDS	3.52	2.17E-52
25	hypothetical protein CDS	3.51	0
26	queT 2 CDS	3.51	1.25E-251
27	emrB 1 CDS	3.50	1.05E-223
28	thiT CDS	3.50	1.23E-294
29	sstT CDS	3.47	4.10E-238
30	yicL 1 CDS	3.45	8.19E-262
31	hypothetical protein CDS	3.44	2.02E-285
32	hypothetical protein CDS	3.42	2.24E-92
33	hypothetical protein CDS	3.41	5.22E-52
34	hypothetical protein CDS	3.41	4.72E-219
35	ISNCY family transposase ISSeq2 CDS	3.40	0
36	bglF 3 CDS	3.34	3.02E-234

37	hypothetical protein CDS	3.34	1.99E-22
38	ydfK CDS	3.33	0
39	hypothetical protein CDS	3.33	2.24E-175
40	plsY CDS	3.29	2.13E-298
41	hypothetical protein CDS	3.29	9.28E-96
42	ycaM 1 CDS	3.28	1.12E-178
43	hypothetical protein CDS	3.28	3.65E-149
44	hypothetical protein CDS	3.27	0
45	hypothetical protein CDS	3.23	0
46	hypothetical protein CDS	3.23	2.61E-61
47	bacF CDS	3.23	7.50E-43
48	nhaK 1 CDS	3.23	3.00E-277
49	glpF CDS	3.20	1.41E-70
50	ygaZ CDS	3.19	1.79E-70
51	hypothetical protein CDS	3.18	9.83E-258
52	hypothetical protein CDS	3.16	1.64E-50
53	glnP CDS	3.15	1.38E-211
54	dtpT CDS	3.15	0
55	appC CDS	3.13	8.50E-89
56	hypothetical protein CDS	3.11	7.73E-194
57	hypothetical protein CDS	3.11	2.20E-165
58	hypothetical protein CDS	3.09	5.99E-82
59	hypothetical protein CDS	3.09	9.42E-74
60	hypothetical protein CDS	3.08	0
61	psiE CDS	3.05	3.74E-155
62	hypothetical protein CDS	3.04	6.62E-19
63	hypothetical protein CDS	3.04	7.50E-255
64	hypothetical protein CDS	3.03	1.79E-201
65	hypothetical protein CDS	3.02	6.80E-141
66	hypothetical protein CDS	3.02	2.54E-148
67	hypothetical protein CDS	3.02	2.09E-176
68	bglF 2 CDS	3.01	2.37E-62
69	cas1 CDS	2.99	2.67E-138
70	znuB CDS	2.98	9.62E-186
71	amt CDS	2.97	3.22E-57
72	fecE CDS	2.97	9.78E-28
73	hypothetical protein CDS	2.97	1.90E-134
74	hsrA 2 CDS	2.97	6.76E-94
75	ycaM 2 CDS	2.97	2.08E-285
76	hypothetical protein CDS	2.94	4.40E-195
77	hypothetical protein CDS	2.93	2.32E-137
78	hypothetical protein CDS	2.93	8.42E-280
79	ykoD 1 CDS	2.90	1.04E-12

80	btuD 19 CDS	2.89	1.68E-144
81	hypothetical protein CDS	2.89	3.26E-168
82	adeP CDS	2.89	5.66E-267
83	hypothetical protein CDS	2.88	6.60E-104
84	hypothetical protein CDS	2.88	4.35E-49
85	hypothetical protein CDS	2.87	2.06E-268
86	folT CDS	2.85	2.16E-286
87	patB 1 CDS	2.84	1.09E-65
88	frlD CDS	2.84	3.82E-223
89	hypothetical protein CDS	2.83	1.27E-225
90	hypothetical protein CDS	2.83	2.65E-210
91	bmr3 2 CDS	2.83	1.47E-123
92	ybhL CDS	2.82	3.13E-269
93	hypothetical protein CDS	2.82	8.96E-264
94	hypothetical protein CDS	2.81	1.40E-13
95	yafV CDS	2.79	2.66E-17
96	hypothetical protein CDS	2.79	3.41E-207
97	rpmB CDS	2.79	1.46E-250
98	hypothetical protein CDS	2.79	1.15E-12
99	hypothetical protein CDS	2.78	6.01E-131
100	hypothetical protein CDS	2.78	6.59E-33
101	hypothetical protein CDS	2.78	1.01E-67
102	ypdE CDS	2.78	7.12E-30
103	lacF 1 CDS	2.78	1.15E-27
104	hypothetical protein CDS	2.77	1.66E-19
105	hypothetical protein CDS	2.77	1.85E-136
106	gla CDS	2.77	1.01E-234
107	hypothetical protein CDS	2.77	5.11E-13
108	lspA CDS	2.76	1.24E-164
109	yycJ CDS	2.75	5.05E-149
110	hypothetical protein CDS	2.75	1.95E-181
111	hypothetical protein CDS	2.75	1.79E-47
112	recD2 2 CDS	2.74	3.76E-175
113	hypothetical protein CDS	2.74	1.02E-228
114	cycA CDS	2.73	2.90E-239
115	hypothetical protein CDS	2.73	7.31E-21
116	hypothetical protein CDS	2.73	1.66E-166
117	hypothetical protein CDS	2.73	1.09E-34
118	hypothetical protein CDS	2.72	5.65E-53
119	hypothetical protein CDS	2.71	1.96E-164
120	hypothetical protein CDS	2.70	1.33E-89
121	zapA CDS	2.70	6.15E-101
122	yhfP CDS	2.66	8.23E-174

123	chbC CDS	2.66	4.82E-157
124	hypothetical protein CDS	2.66	1.18E-85
125	hypothetical protein CDS	2.65	8.73E-16
126	hypothetical protein CDS	2.64	1.68E-43
127	yfcA CDS	2.64	2.83E-52
128	mdtG CDS	2.64	2.49E-183
129	hypothetical protein CDS	2.64	4.79E-149
130	lysP 2 CDS	2.64	4.63E-164
131	rbsR CDS	2.63	5.88E-149
132	dedA CDS	2.63	4.82E-240
133	hypothetical protein CDS	2.63	6.58E-165
134	hypothetical protein CDS	2.63	1.67E-36
135	csp CDS	2.63	2.91E-191
136	rex 1 CDS	2.63	1.07E-95
137	hypothetical protein CDS	2.62	7.33E-44
138	hypothetical protein CDS	2.62	4.22E-210
139	ytlR CDS	2.62	1.22E-231
140	hypothetical protein CDS	2.62	4.21E-12
141	metQ 4 CDS	2.61	5.12E-19
142	hypothetical protein CDS	2.61	5.11E-199
143	queT 1 CDS	2.61	1.52E-256
144	Calcium-transporting ATPase 1 CDS	2.60	3.00E-61
145	hypothetical protein CDS	2.60	6.29E-71
146	hypothetical protein CDS	2.60	1.30E-181
147	hypothetical protein CDS	2.59	2.52E-119
148	hypothetical protein CDS	2.57	4.91E-229
149	hypothetical protein CDS	2.57	8.38E-207
150	tadA 2 CDS	2.55	5.90E-79
151	murB CDS	2.54	3.27E-154
152	oppC 1 CDS	2.53	1.48E-226
153	hypothetical protein CDS	2.53	8.04E-126
154	hypothetical protein CDS	2.53	5.19E-176
155	pyrR CDS	2.53	2.52E-68
156	hypothetical protein CDS	2.53	1.38E-143
157	hypothetical protein CDS	2.52	1.72E-149
158	glnM CDS	2.52	2.45E-134
159	ulaA 1 CDS	2.52	3.82E-59
160	hypothetical protein CDS	2.51	4.79E-20
161	cydB 1 CDS	2.51	7.60E-29
162	ypdF 1 CDS	2.51	3.59E-49
163	hypothetical protein CDS	2.51	3.44E-06
164	apu CDS	2.50	2.71E-209
165	hadL CDS	2.49	1.43E-52

166	yveA CDS	2.48	9.55E-189
167	hypothetical protein CDS	2.48	0.00078105
168	hypothetical protein CDS	2.48	6.78E-18
169	hypothetical protein CDS	2.47	2.88E-96
170	hypothetical protein CDS	2.47	1.70E-152
171	malL 1 CDS	2.46	6.07E-39
172	hypothetical protein CDS	2.45	3.16E-126
173	hypothetical protein CDS	2.45	1.04E-91
174	hypothetical protein CDS	2.45	1.31E-65
175	hypothetical protein CDS	2.43	9.38E-130
176	hypothetical protein CDS	2.43	1.56E-06
177	ribZ 6 CDS	2.43	3.67E-120
178	arnT CDS	2.42	1.70E-37
179	treP CDS	2.42	9.70E-16
180	bceA 1 CDS	2.41	2.64E-121
181	hypothetical protein CDS	2.41	4.81E-131
182	gbuB CDS	2.40	9.32E-69
183	hypothetical protein CDS	2.40	2.70E-164
184	potB CDS	2.40	2.91E-121
185	hypothetical protein CDS	2.39	2.21E-37
186	hypothetical protein CDS	2.39	2.02E-171
187	hypothetical protein CDS	2.39	1.31E-147
188	hypothetical protein CDS	2.38	1.28E-67
189	hypothetical protein CDS	2.37	1.67E-68
190	hypothetical protein CDS	2.37	2.09E-146
191	hypothetical protein CDS	2.37	4.75E-140
192	mntB CDS	2.37	1.24E-18
193	hypothetical protein CDS	2.37	3.69E-63
194	hypothetical protein CDS	2.36	1.53E-17
195	ycaC CDS	2.35	2.03E-151
196	iolT 2 CDS	2.34	9.18E-127
197	hypothetical protein CDS	2.34	2.81E-100
198	hypothetical protein CDS	2.34	2.52E-142
199	mscL CDS	2.33	6.13E-105
200	hypothetical protein CDS	2.32	6.28E-40
201	hypothetical protein CDS	2.30	5.51E-75
202	amtB CDS	2.30	4.88E-12
203	hypothetical protein CDS	2.29	2.78E-152
204	hypothetical protein CDS	2.29	1.41E-84
205	hypothetical protein CDS	2.29	4.27E-08
206	hypothetical protein CDS	2.29	1.24E-111
207	btuD 3 CDS	2.29	6.79E-78
208	fni CDS	2.29	1.43E-126

209	hypothetical protein CDS	2.29	3.68E-36
210	degA 1 CDS	2.29	2.47E-133
211	hypothetical protein CDS	2.28	1.58E-76
212	hypothetical protein CDS	2.28	4.46E-125
213	hypothetical protein CDS	2.27	1.27E-65
214	galK 2 CDS	2.26	1.22E-70
215	hypothetical protein CDS	2.26	4.58E-21
216	ribZ 3 CDS	2.26	1.06E-129
217	fas6 CDS	2.25	2.52E-158
218	glcU CDS	2.24	2.61E-145
219	hypothetical protein CDS	2.24	1.06E-35
220	hypothetical protein CDS	2.24	5.25E-147
221	gbuC CDS	2.24	2.29E-50
222	hypothetical protein CDS	2.24	1.22E-84
223	hypothetical protein CDS	2.23	8.44E-81
224	hypothetical protein CDS	2.23	7.46E-07
225	hypothetical protein CDS	2.23	4.00E-93
226	hypothetical protein CDS	2.23	3.92E-112
227	hypothetical protein CDS	2.23	2.65E-146
228	yicL 2 CDS	2.22	7.22E-117
229	rhaB 2 CDS	2.22	3.80E-16
230	ycaM 3 CDS	2.21	6.71E-133
231	obg CDS	2.20	3.99E-171
232	hmo CDS	2.20	1.17E-85
233	hypothetical protein CDS	2.19	4.74E-125
234	folA CDS	2.19	8.44E-143
235	rnhA 1 CDS	2.18	1.04E-89
236	mleS CDS	2.18	7.09E-67
237	hypothetical protein CDS	2.18	2.26E-20
238	secG CDS	2.18	1.66E-141
239	hypothetical protein CDS	2.18	4.58E-22
240	hypothetical protein CDS	2.18	1.24E-137
241	melA CDS	2.18	1.32E-105
242	hypothetical protein CDS	2.17	8.58E-66
243	Putative glutamine amidotransferase CDS	2.17	1.59E-111
244	hypothetical protein CDS	2.16	1.46E-25
245	pyrP CDS	2.16	1.89E-139
246	mprF CDS	2.15	2.15E-155
247	clcA 2 CDS	2.14	2.04E-156
248	hypothetical protein CDS	2.14	2.89E-30
249	hypothetical protein CDS	2.14	3.89E-80
250	bmr3 1 CDS	2.14	2.69E-115
251	nhaK 2 CDS	2.13	5.35E-120

252	hypothetical protein CDS	2.13	6.49E-119
253	hypothetical protein CDS	2.13	4.84E-11
254	hypothetical protein CDS	2.13	1.42E-48
255	hypothetical protein CDS	2.13	1.03E-134
256	hypothetical protein CDS	2.12	1.21E-71
257	zosA 1 CDS	2.12	1.14E-16
258	hypothetical protein CDS	2.12	1.32E-120
259	hypothetical protein CDS	2.12	4.73E-36
260	hypothetical protein CDS	2.11	1.31E-71
261	uup CDS	2.11	4.95E-48
262	murJ 1 CDS	2.11	6.92E-158
263	zur CDS	2.10	2.46E-48
264	hypothetical protein CDS	2.10	1.27E-128
265	IS66 family transposase ISSwo2 CDS	2.10	5.42E-46
266	recQ 1 CDS	2.10	1.56E-125
267	hypothetical protein CDS	2.10	5.61E-90
268	hypothetical protein CDS	2.09	2.22E-11
269	hypothetical protein CDS	2.09	5.28E-22
270	ykoE CDS	2.09	0.00154146
271	yidA 1 CDS	2.09	1.73E-89
272	atzA CDS	2.09	1.71E-165
273	kup CDS	2.08	1.67E-123
274	comFA CDS	2.08	2.25E-16
275	hypothetical protein CDS	2.08	2.68E-23
276	hypothetical protein CDS	2.08	2.25E-12
277	ftsW CDS	2.07	2.30E-111
278	hypothetical protein CDS	2.07	3.43E-87
279	hypothetical protein CDS	2.06	1.89E-106
280	kduD CDS	2.06	1.13E-130
281	hypothetical protein CDS	2.06	7.89E-92
282	hypothetical protein CDS	2.05	2.27E-24
283	putative ABC transporter ATP-binding protein CDS	2.05	4.35E-07
284	hypothetical protein CDS	2.05	0.00013315
285	czcD CDS	2.05	2.01E-132
286	hypothetical protein CDS	2.05	7.32E-67
287	ymfD CDS	2.05	8.30E-14
288	hypothetical protein CDS	2.04	8.00E-117
289	hypothetical protein CDS	2.03	1.52E-152
290	hypothetical protein CDS	2.03	0.00019077
291	lolD 1 CDS	2.03	3.45E-46
292	lysP 1 CDS	2.03	3.11E-23
293	rnhA 2 CDS	2.03	4.36E-128
294	bglH 2 CDS	2.03	5.01E-28

295	hypothetical protein CDS	2.03	1.15E-88
296	minJ CDS	2.02	6.21E-143
297	rbsK/rbiA CDS	2.02	1.22E-92
298	hypothetical protein CDS	2.02	1.15E-42
299	hypothetical protein CDS	2.02	1.20E-14
300	ribZ 8 CDS	2.02	1.65E-32
301	smc 3 CDS	2.02	8.19E-24
302	hypothetical protein CDS	2.01	1.34E-60
303	licB CDS	2.01	1.04E-74
304	hypothetical protein CDS	2.00	1.00E-91
305	hypothetical protein CDS	2.00	7.19E-140
306	hypothetical protein CDS	2.00	2.38E-48
307	hypothetical protein CDS	2.00	1.07E-50
308	ssuB CDS	1.99	1.12E-07
309	lacD 1 CDS	-1.90	9.38E-35
310	IS200/IS605 family transposase ISLhe65 CDS	-1.90	1.22E-36
311	hypothetical protein CDS	-1.91	4.49E-15
312	hypothetical protein CDS	-1.91	9.88E-67
313	hypothetical protein CDS	-1.91	1.76E-28
314	hypothetical protein CDS	-1.91	8.95E-73
315	fabG 2 CDS	-1.93	8.12E-84
316	hypothetical protein CDS	-1.93	4.79E-69
317	hypothetical protein CDS	-1.93	1.75E-10
318	fabG2 CDS	-1.93	6.94E-85
319	hypothetical protein CDS	-1.94	5.86E-43
320	hypothetical protein CDS	-1.94	2.06E-44
321	hypothetical protein CDS	-1.94	0.00400341
322	cinA CDS	-1.94	1.37E-77
323	tpiA CDS	-1.95	4.28E-71
324	hypothetical protein CDS	-1.95	7.45E-39
325	rplW CDS	-1.95	1.60E-132
326	fabZ 1 CDS	-1.95	3.51E-57
327	hypothetical protein CDS	-1.95	2.10E-13
328	regX3 CDS	-1.96	1.11E-42
329	hypothetical protein CDS	-1.96	2.53E-49
330	Putative bifunctional phosphatase/peptidyl-prolyl cis-trans isomerase	-1.97	1.94E-83
331	gmk 1 CDS	-1.97	2.79E-76
332	hypothetical protein CDS	-1.98	4.63E-102
333	nrdE1 CDS	-1.98	8.51E-101
334	hypothetical protein CDS	-1.98	2.06E-52
335	rplGA CDS	-1.98	1.73E-67
336	appA CDS	-1.99	2.96E-122
337	sasA 4 CDS	-1.99	6.82E-43

338	hypothetical protein CDS	-1.99	3.66E-43
339	fabF CDS	-2.00	2.18E-86
340	efp 1 CDS	-2.01	6.67E-129
341	hypothetical protein CDS	-2.01	1.56E-32
342	lacB CDS	-2.01	1.72E-34
343	hypothetical protein CDS	-2.01	9.60E-34
344	hypothetical protein CDS	-2.02	3.11E-111
345	rplB CDS	-2.02	1.29E-143
346	ychF CDS	-2.02	1.17E-110
347	adhE CDS	-2.02	4.70E-98
348	hypothetical protein CDS	-2.02	4.58E-16
349	hypothetical protein CDS	-2.03	2.39E-77
350	pstB3 2 CDS	-2.03	7.96E-101
351	recU CDS	-2.03	1.03E-66
352	rmlC 1 CDS	-2.03	6.44E-98
353	rpoE CDS	-2.03	4.61E-102
354	hypothetical protein CDS	-2.04	4.23E-22
355	polA CDS	-2.05	1.62E-101
356	hypothetical protein CDS	-2.05	8.42E-80
357	hypothetical protein CDS	-2.05	1.27E-47
358	lacA 2 CDS	-2.06	5.28E-26
359	rbgA CDS	-2.06	2.06E-68
360	gtf1 1 CDS	-2.07	4.94E-62
361	srrA 1 CDS	-2.07	3.44E-107
362	hypothetical protein CDS	-2.08	2.79E-41
363	hypothetical protein CDS	-2.08	1.51E-131
364	hypothetical protein CDS	-2.09	4.67E-61
365	uvrC CDS	-2.09	5.35E-91
366	ndk CDS	-2.09	6.55E-105
367	cas2 CDS	-2.09	1.76E-12
368	gtf1 2 CDS	-2.10	3.11E-66
369	ffh CDS	-2.10	6.26E-96
370	relA CDS	-2.10	2.16E-131
371	nagB CDS	-2.10	3.24E-99
372	yidA 3 CDS	-2.11	2.72E-69
373	dacA 1 CDS	-2.11	1.45E-131
374	hypothetical protein CDS	-2.11	6.08E-61
375	ugpB 1 CDS	-2.12	4.21E-164
376	metQ 3 CDS	-2.12	1.20E-61
377	ctpA CDS	-2.12	7.65E-99
378	hypothetical protein CDS	-2.12	1.28E-79
379	hypothetical protein CDS	-2.12	8.71E-45
380	rpoA CDS	-2.13	6.25E-162

381	bceA 2 CDS	-2.13	2.26E-79
382	hypothetical protein CDS	-2.13	1.67E-76
383	hypothetical protein CDS	-2.13	3.90E-144
384	hypothetical protein CDS	-2.14	1.39E-82
385	ywqD CDS	-2.15	5.08E-127
386	hypothetical protein CDS	-2.15	3.76E-45
387	frwD CDS	-2.15	0.00014857
388	hypothetical protein CDS	-2.15	2.71E-28
389	hypothetical protein CDS	-2.15	2.27E-160
390	pepDA 1 CDS	-2.15	3.32E-128
391	serS CDS	-2.16	4.62E-147
392	hypothetical protein CDS	-2.16	3.48E-37
393	php CDS	-2.16	4.66E-16
394	hypothetical protein CDS	-2.16	8.37E-76
395	pgk CDS	-2.17	6.05E-95
396	gpmA 3 CDS	-2.18	3.75E-165
397	srrA 3 CDS	-2.18	1.14E-108
398	hypothetical protein CDS	-2.18	2.20E-11
399	udk CDS	-2.18	9.50E-93
400	hypothetical protein CDS	-2.19	9.61E-71
401	eno2 CDS	-2.19	5.42E-113
402	hypothetical protein CDS	-2.19	2.41E-32
403	rpoZ CDS	-2.19	2.42E-51
404	xerC 3 CDS	-2.19	4.94E-71
405	dppE 4 CDS	-2.19	1.45E-143
406	rfbA 1 CDS	-2.19	1.08E-130
407	rpsT CDS	-2.20	2.27E-135
408	kbaY CDS	-2.20	2.36E-36
409	yhfS CDS	-2.21	1.46E-15
410	hypothetical protein CDS	-2.22	5.66E-10
411	putative RNA methyltransferase CDS	-2.23	3.82E-117
412	recR CDS	-2.23	2.28E-97
413	hypothetical protein CDS	-2.24	1.46E-138
414	mtnB CDS	-2.24	3.43E-21
415	hypothetical protein CDS	-2.24	3.89E-11
416	rpsM CDS	-2.24	1.11E-108
417	hypothetical protein CDS	-2.25	5.64E-13
418	hypothetical protein CDS	-2.25	1.14E-31
419	hypothetical protein CDS	-2.25	2.72E-46
420	grpE CDS	-2.25	5.05E-107
421	hypothetical protein CDS	-2.25	1.68E-119
422	hypothetical protein CDS	-2.25	1.09E-133
423	oppA 1 CDS	-2.26	7.36E-171

424	fpaP 1 CDS	-2.26	2.89E-92
425	soj CDS	-2.26	1.04E-105
426	rpsK CDS	-2.27	2.46E-125
427	rplX CDS	-2.27	9.41E-107
428	acpP 1 CDS	-2.27	3.84E-68
429	hypothetical protein CDS	-2.28	4.09E-43
430	pmpR CDS	-2.28	4.92E-192
431	rpsS CDS	-2.29	6.97E-162
432	DegV domain-containing protein CDS	-2.29	2.29E-147
433	hypothetical protein CDS	-2.30	4.91E-147
434	hypothetical protein CDS	-2.30	3.01E-20
435	hypothetical protein CDS	-2.30	1.83E-44
436	ccpA CDS	-2.30	6.97E-119
437	ligA 1 CDS	-2.30	1.52E-12
438	def CDS	-2.31	1.86E-121
439	nusA CDS	-2.32	2.55E-124
440	hypothetical protein CDS	-2.33	2.97E-93
441	celA 4 CDS	-2.33	2.33E-24
442	hypothetical protein CDS	-2.33	1.30E-29
443	cynR CDS	-2.34	1.20E-105
444	hypothetical protein CDS	-2.34	1.09E-167
445	valS CDS	-2.35	3.02E-113
446	clpB CDS	-2.35	5.20E-112
447	hypothetical protein CDS	-2.35	7.15E-13
448	hypothetical protein CDS	-2.36	1.11E-124
449	hypothetical protein CDS	-2.37	2.06E-24
450	hypothetical protein CDS	-2.38	9.76E-72
451	prfB CDS	-2.39	6.13E-120
452	Putative universal stress protein CDS	-2.39	5.24E-171
453	hypothetical protein CDS	-2.39	1.38E-71
454	xerC 5 CDS	-2.40	4.30E-104
455	hypothetical protein CDS	-2.40	3.98E-127
456	pksC CDS	-2.41	3.14E-120
457	Nucleoid-associated protein CDS	-2.42	8.45E-101
458	hypothetical protein CDS	-2.42	6.13E-17
459	hypothetical protein CDS	-2.43	1.39E-14
460	hypothetical protein CDS	-2.43	1.72E-63
461	hypothetical protein CDS	-2.43	2.67E-53
462	hypothetical protein CDS	-2.44	2.05E-08
463	Putative universal stress protein CDS	-2.45	2.62E-162
464	hypothetical protein CDS	-2.45	1.84E-156
465	rplV CDS	-2.46	5.43E-188
466	hypothetical protein CDS	-2.46	2.21E-98

467	rcsC 2 CDS	-2.47	4.54E-175
468	lacD 2 CDS	-2.47	1.40E-174
469	hypothetical protein CDS	-2.48	0.00124017
470	brpA 2 CDS	-2.48	8.25E-202
471	hypothetical protein CDS	-2.48	1.38E-48
472	hpf CDS	-2.49	4.31E-194
473	hypothetical protein CDS	-2.49	3.11E-54
474	noc CDS	-2.49	2.21E-125
475	hypothetical protein CDS	-2.50	4.17E-102
476	lysN CDS	-2.50	4.26E-246
477	rmlD CDS	-2.50	4.04E-212
478	hypothetical protein CDS	-2.51	4.85E-18
479	groL CDS	-2.52	3.75E-194
480	accB CDS	-2.52	1.11E-73
481	psaA CDS	-2.52	1.33E-62
482	hypothetical protein CDS	-2.54	1.13E-59
483	hypothetical protein CDS	-2.54	6.05E-05
484	hypothetical protein CDS	-2.54	5.61E-41
485	yybR CDS	-2.54	4.91E-75
486	lacR 1 CDS	-2.56	1.09E-28
487	hypothetical protein CDS	-2.56	9.75E-165
488	spo0J CDS	-2.57	2.27E-143
489	hypothetical protein CDS	-2.58	2.47E-174
490	hypothetical protein CDS	-2.58	1.34E-199
491	hypothetical protein CDS	-2.59	3.45E-35
492	mngA CDS	-2.59	1.64E-07
493	tal 2 CDS	-2.60	3.78E-193
494	hypothetical protein CDS	-2.60	1.12E-36
495	rcsC 4 CDS	-2.60	6.29E-159
496	recA 2 CDS	-2.60	6.54E-190
497	hypothetical protein CDS	-2.60	3.90E-132
498	TelA-like protein CDS	-2.61	1.14E-171
499	rpsB CDS	-2.61	5.50E-174
500	naiP CDS	-2.61	6.55E-211
501	hypothetical protein CDS	-2.62	1.79E-170
502	nagZ CDS	-2.62	3.42E-78
503	rpsC CDS	-2.62	6.20E-192
504	ldh 3 CDS	-2.63	4.77E-216
505	hypothetical protein CDS	-2.63	6.07E-161
506	hit CDS	-2.64	4.75E-106
507	immR CDS	-2.64	5.24E-93
508	clpE CDS	-2.64	6.32E-130
509	rmlB 1 CDS	-2.65	3.65E-253

510	hypothetical protein CDS	-2.66	2.72E-19
511	yceM CDS	-2.66	3.35E-192
512	hypothetical protein CDS	-2.67	3.10E-78
513	rpiA CDS	-2.68	1.89E-101
514	hypothetical protein CDS	-2.68	1.04E-179
515	hypothetical protein CDS	-2.71	1.03E-25
516	hypothetical protein CDS	-2.71	1.08E-92
517	hypothetical protein CDS	-2.74	2.05E-122
518	hypothetical protein CDS	-2.74	9.84E-113
519	dnaK CDS	-2.75	8.22E-248
520	hypothetical protein CDS	-2.76	4.05E-111
521	hypothetical protein CDS	-2.76	6.49E-41
522	rplN CDS	-2.77	1.13E-154
523	hypothetical protein CDS	-2.77	2.49E-08
524	hypothetical protein CDS	-2.78	2.64E-37
525	rpmC CDS	-2.78	2.84E-163
526	hypothetical protein CDS	-2.79	6.59E-17
527	hypothetical protein CDS	-2.80	5.09E-75
528	hypothetical protein CDS	-2.80	4.67E-146
529	hypothetical protein CDS	-2.81	1.49E-195
530	mntA CDS	-2.84	2.22E-89
531	hypothetical protein CDS	-2.85	1.28E-113
532	D-2-hydroxyisocaproate dehydrogenase CDS	-2.85	1.45E-216
533	rpsQ CDS	-2.87	1.70E-243
534	hypothetical protein CDS	-2.90	1.48E-163
535	gltX CDS	-2.90	6.57E-302
536	hypothetical protein CDS	-2.92	6.19E-304
537	hypothetical protein CDS	-2.92	1.27E-241
538	Nitronate monooxygenase CDS	-2.93	3.53E-162
539	hypothetical protein CDS	-2.95	2.83E-69
540	hypothetical protein CDS	-2.98	2.92E-79
541	hypothetical protein CDS	-2.98	2.63E-286
542	hypothetical protein CDS	-2.99	9.93E-211
543	frr CDS	-2.99	1.61E-240
544	hypothetical protein CDS	-3.01	1.84E-138
545	hypothetical protein CDS	-3.01	2.86E-237
546	rplP CDS	-3.02	1.93E-248
547	hypothetical protein CDS	-3.07	5.32E-40
548	hypothetical protein CDS	-3.09	6.32E-273
549	hypothetical protein CDS	-3.11	2.84E-134
550	hypothetical protein CDS	-3.17	2.27E-79
551	degA 2 CDS	-3.19	1.12E-76
552	hypothetical protein CDS	-3.20	4.13E-269

553	hypothetical protein CDS	-3.21	2.51E-151
554	hypothetical protein CDS	-3.21	9.49E-153
555	hypothetical protein CDS	-3.24	1.82E-199
556	yqeY CDS	-3.26	2.18E-214
557	hypothetical protein CDS	-3.28	1.20E-218
558	hypothetical protein CDS	-3.29	7.26E-203
559	prsA 1 CDS	-3.32	0
560	rpsU CDS	-3.35	5.06E-229
561	fabH CDS	-3.36	0
562	hypothetical protein CDS	-3.36	1.30E-57
563	hypothetical protein CDS	-3.40	1.44E-233
564	hypothetical protein CDS	-3.42	6.25E-147
565	hypothetical protein CDS	-3.43	0
566	hypothetical protein CDS	-3.51	2.80E-91
567	hypothetical protein CDS	-3.56	5.34E-146
568	hypothetical protein CDS	-3.59	3.22E-61
569	hypothetical protein CDS	-3.64	8.27E-81
570	hypothetical protein CDS	-3.71	4.94E-205
571	hypothetical protein CDS	-3.75	5.23E-214
572	acpP 2 CDS	-3.77	7.38E-303
573	hypothetical protein CDS	-3.86	0
574	tig CDS	-3.99	0
575	hypothetical protein CDS	-4.04	3.01E-185
576	hypothetical protein CDS	-4.75	0
577	hypothetical protein CDS	-5.99	0
	**Lactiplantibacillus plantarum Lp-115**		
	**Name**	**Differential Expression Log2 Ratio**	**Differential Expression p-value**
1	Transcriptional regulator, PadR family CDS	2.39	8.22E-27
2	Diaminohydroxyphosphoribosylaminopyrimidine deaminase (EC 3.5.4.26) / 5-amino-6-(5-phosphoribosylamino)uracil reductase (EC 1.1.1.193) CDS	2.00	2.75E-37
3	Di-tripeptide/cation symporter DtpT CDS	1.97	1.18E-14
4	Permease of the drug/metabolite transporter (DMT) superfamily CDS	1.94	2.77E-10
5	Riboflavin synthase eubacterial/eukaryotic (EC 2.5.1.9) CDS	1.92	3.49E-32
6	Cold shock protein of CSP family CDS	1.91	7.86E-09
7	Cold shock protein of CSP family CDS	1.91	1.79E-11
8	hypothetical protein CDS	-1.91	4.03E-26
9	prophage Lp2 protein 45 CDS	-1.93	1.22E-20
10	hypothetical protein CDS	-1.93	2.27E-23
11	integral membrane protein CDS	-1.95	3.44E-19
12	hypothetical protein CDS	-1.95	2.65E-12
13	Putative prophage protein (ps3) CDS	-1.98	1.41E-30
14	hypothetical protein CDS	-1.99	1.85E-25

15	hypothetical protein CDS	-1.99	3.07E-22
16	Heat shock protein GrpE CDS	-1.99	1.41E-12
17	hypothetical protein CDS	-2.02	6.20E-39
18	prophage Lp1 protein 45 CDS	-2.03	2.01E-24
19	prophage Lp1 protein 48 CDS	-2.07	2.29E-28
20	hypothetical protein CDS	-2.13	5.27E-16
21	hypothetical protein CDS	-2.15	1.02E-37
22	integral membrane protein CDS	-2.16	4.97E-11
23	hypothetical protein CDS	-2.16	2.94E-25
24	hypothetical protein CDS	-2.19	2.00E-39
25	Exonuclease SbcC CDS	-2.22	5.62E-44
26	hypothetical protein CDS	-2.23	5.77E-29
27	Transcriptional regulator CDS	-2.23	6.62E-16
28	hypothetical protein CDS	-2.24	8.23E-42
29	Chaperone protein DnaK CDS	-2.25	3.45E-20
30	hypothetical protein CDS	-2.26	3.73E-52
31	hypothetical protein CDS	-2.28	3.29E-21
32	prophage Lp1 protein 5 CDS	-2.29	2.11E-32
33	Hypothetical protein SAV1845 CDS	-2.30	1.89E-48
34	DNA-cytosine methyltransferase (EC 2.1.1.37) CDS	-2.30	4.38E-50
35	Predicted transcriptional regulators CDS	-2.34	1.09E-40
36	hypothetical protein CDS	-2.35	1.41E-41
37	Replication initiation protein A CDS	-2.35	3.72E-50
38	PTS IIA-like nitrogen-regulatory protein PtsN CDS	-2.42	3.09E-39
39	hypothetical protein CDS	-2.44	8.67E-33
40	Transcription antiterminator, BglG family CDS	-2.45	3.01E-58
41	prophage Lp2 protein 32 CDS	-2.45	7.07E-27
42	hypothetical protein CDS	-2.47	1.84E-62
43	hypothetical protein CDS	-2.48	1.28E-36
44	Phage transcriptional activator RinA CDS	-2.52	3.63E-62
45	hypothetical protein CDS	-2.58	9.37E-42
46	cell surface protein precursor CDS	-2.59	7.18E-39
47	hypothetical protein CDS	-2.59	8.91E-24
48	cell surface protein precursor CDS	-2.59	1.90E-15
49	hypothetical protein CDS	-2.67	1.08E-44
50	FIG00754769: hypothetical protein CDS	-2.69	6.63E-28
51	extracellular protein CDS	-2.70	8.48E-59
52	hypothetical protein CDS	-2.76	4.64E-74
53	Prophage Lp1 protein 19 CDS	-2.79	1.25E-60
54	DNA topoisomerase III (EC 5.99.1.2) CDS	-2.80	6.75E-41
55	hypothetical protein CDS	-2.81	7.58E-38
56	putative plasmid partition protein CDS	-2.82	1.95E-51
57	immunity repressor protein (phage-related protein) CDS	-2.82	2.68E-60

58	hypothetical protein CDS	-2.84	3.32E-25
59	hypothetical protein CDS	-2.89	3.66E-42
60	hypothetical protein CDS	-2.92	2.93E-50
61	prophage Lp2 protein 25 CDS	-2.92	1.99E-43
62	unknown CDS	-2.94	1.91E-31
63	FIG00745688: hypothetical protein CDS	-2.98	4.80E-53
64	putative antirepressor protein CDS	-2.99	5.48E-72
65	DNA-damage-inducible gene CDS	-3.09	2.12E-57
66	cell surface protein precursor CDS	-3.12	1.66E-49
67	hypothetical protein CDS	-3.12	6.00E-51
68	hypothetical protein CDS	-3.12	1.18E-37
69	hypothetical protein CDS	-3.12	7.26E-59
70	hypothetical protein CDS	-3.14	5.33E-64
71	prophage Lp1 protein 17 CDS	-3.16	3.06E-76
72	hypothetical protein CDS	-3.18	6.47E-44
73	prophage Lp2 protein 12 CDS	-3.22	2.87E-53
74	Recombinational DNA repair protein RecT (prophage associated) CDS	-3.27	1.25E-71
75	prophage Lp2 protein 11 CDS	-3.27	4.36E-59
76	hypothetical protein CDS	-3.29	2.88E-66
77	hypothetical protein CDS	-3.32	3.41E-74
78	hypothetical protein CDS	-3.35	1.74E-56
79	hypothetical protein CDS	-3.47	4.24E-92
80	hypothetical protein CDS	-3.47	5.89E-78
81	hypothetical protein CDS	-3.50	1.22E-80
82	hypothetical protein CDS	-3.53	1.58E-60
83	FIG00742586: hypothetical protein CDS	-3.54	1.29E-52
84	hypothetical protein CDS	-3.61	7.79E-56
85	hyp.protein CDS replisome organizer	-3.61	1.77E-102
86	hypothetical protein CDS	-3.64	7.68E-77
87	Nickase CDS	-3.70	1.52E-78
88	DNA replication protein CDS	-3.92	1.62E-110
